# Prolonged exciton lifetime *via* conjugation-length engineering in M-series acceptors for 19.39% efficiency polymer solar cells

**DOI:** 10.1093/nsr/nwaf537

**Published:** 2025-11-29

**Authors:** Wenxiong Shen, Xiaoying Xiong, Dongdong Cai, Li Liu, Junlu Lin, Shuo Wan, Jin-Yun Wang, Yi Li, Yunlong Ma, Huiting Fu, Chunfeng Zhang, Qingdong Zheng

**Affiliations:** State Key Laboratory of Coordination Chemistry, College of Engineering and Applied Sciences, Nanjing University, Nanjing 210023, China; State Key Laboratory of Coordination Chemistry, College of Engineering and Applied Sciences, Nanjing University, Nanjing 210023, China; State Key Laboratory of Structural Chemistry, Fujian Institute of Research on the Structure of Matter, Chinese Academy of Sciences, Fuzhou 350002, China; State Key Laboratory of Structural Chemistry, Fujian Institute of Research on the Structure of Matter, Chinese Academy of Sciences, Fuzhou 350002, China; National Laboratory of Solid State Microstructures, School of Physics, and Collaborative Innovation Center for Advanced Microstructures, Nanjing University, Nanjing 210093, China; State Key Laboratory of Coordination Chemistry, College of Engineering and Applied Sciences, Nanjing University, Nanjing 210023, China; State Key Laboratory of Structural Chemistry, Fujian Institute of Research on the Structure of Matter, Chinese Academy of Sciences, Fuzhou 350002, China; State Key Laboratory of Coordination Chemistry, College of Engineering and Applied Sciences, Nanjing University, Nanjing 210023, China; State Key Laboratory of Structural Chemistry, Fujian Institute of Research on the Structure of Matter, Chinese Academy of Sciences, Fuzhou 350002, China; State Key Laboratory of Coordination Chemistry, College of Engineering and Applied Sciences, Nanjing University, Nanjing 210023, China; National Laboratory of Solid State Microstructures, School of Physics, and Collaborative Innovation Center for Advanced Microstructures, Nanjing University, Nanjing 210093, China; State Key Laboratory of Coordination Chemistry, College of Engineering and Applied Sciences, Nanjing University, Nanjing 210023, China

**Keywords:** nonfullerene acceptors, polymer solar cells, exciton binding energy, exciton lifetime, thermal diffusion coefficients, stability

## Abstract

Developing non-fullerene acceptors (NFAs) that combine high device efficiency with superior stability remains a significant challenge. Based on M-series acceptors featuring an acceptor-donor-acceptor (A-D-A)-type framework, we report two dimerized NFAs (DM-8F and DM-8Cl) containing different halogen atoms in their terminal groups. Compared to the small-molecule acceptor M68, both dimerized acceptors exhibit increased glass transition temperatures and enlarged dielectric constants. The choice of halogen atoms in the terminal groups significantly affects their π–π-packing distances, exciton diffusion lengths, and ultimately, photovoltaic performance. Owing to enhanced charge transport, reduced exciton binding energy, and extended exciton diffusion length, DM-8F achieves an efficiency of 19.39% (certified at 19.20%) in small-area polymer solar cells (PSCs) and 15.72% in minimodules with an effective area of 11.09 cm^2^. These efficiencies are the highest reported to date among all A-D-A-type NFAs. Moreover, DM-8F–based devices exhibit significantly improved thermal and photostability compared to those based on M68.

## Teaser

This work presents an A-D-A-type nonfullerene acceptor featuring an extended conjugation length and enhanced exciton lifetime, which enables stable polymer solar cells with a certified efficiency of 19.20%.

## INTRODUCTION

Polymer solar cells (PSCs) are photovoltaic devices capable of converting light energy into electrical energy by utilizing a blend of organic semiconducting electron donor and acceptor materials. Compared to traditional silicon-based solar cells, PSCs offer several advantages, including greater semitransparency, reduced weight, enhanced flexibility and stretchability, and the potential for large-area printing during manufacturing [1–6]. As a result, PSCs hold significant promise for applications in renewable energy, particularly in wearable optoelectronic devices [1,7,8]. However, for practical applications, further improvements in their power conversion efficiencies (PCEs) and stability are required.

Over the past decade, the development of new non-fullerene acceptor (NFA) materials has been at the forefront of research in organic photovoltaics [9–14]. Notably, the ITIC acceptor, featuring an acceptor-donor-acceptor (A-D-A)-type molecular architecture pioneered by Zhan and colleagues in 2015, marked a significant breakthrough by demonstrating device performance comparable to that of conventional fullerene-based acceptors. This advancement propelled the field of PSCs into a new era of research and development [15]. Since this seminal work, a significant number of ITIC-derived NFAs have been synthesized, leading to PSCs that achieved PCEs of ∼14% by 2018 [16–19]. Subsequently, Zou *et al.* incorporated an electron-deficient (A′) unit at the center of the fused-ring structure of the A-D-A molecule, resulting in the formation of an A-DA′D-A-type structure (Y6). Binary PSCs based on Y6 achieved an initial PCE of up to 15.7%, representing a significant advancement in the pursuit of high-efficiency PSCs [20]. Following this, efforts have focused on chemical modifications of Y6 to further enhance the PCE of PSCs. Several modified versions of Y6 have been developed, leading to high-performance binary PSCs with PCEs ranging from 16.1% to 19.1% [6,21–30]. Simultaneously, with the aid of a ternary blending strategy, the highest PCE based on these A-DA'D-A-structured Y6-derived NFAs was further increased to ∼20% [31,32]. In an effort to enhance the thermal stability of monomeric Y6-derived NFAs, dimerized acceptors (DAs) characterized by extended molecular backbones and monodisperse molecular weights have been developed by linking two banana-shaped Y6-derived acceptors. PSCs based on these DAs achieved both good stability and high efficiencies of up to 19% [33–42]. The widely recognized performance of devices based on Y6-derived acceptors (including DAs) has motivated researchers to investigate the fundamental molecular design principles underlying Y6. It is often assumed that strong electron-withdrawing aromatic cores (A′), such as benzothiadiazole and benzotriazole, along with a banana-shaped (curved) molecular structure, are two fundamental design criteria for high-performance NFAs [43–45]. To date, it remains rare for NFAs (including DAs) to achieve comparable or higher PCEs than Y-series acceptors without incorporating a banana-shaped backbone and an electron-deficient unit in the core.

Recently, our group developed a new class of A-D-A-type NFAs (i.e. M-series acceptors) by utilizing a linear-shaped, electron-rich heteroheptacene donor core. This core was constructed by substituting all *sp*^3^-hybridized carbon atoms with *sp*^2^-hybridized nitrogen atoms [46–51]. By controlling π–π stacking and molecular orientation through the neighboring side chains on the heteroheptacene donor core, the resulting M-series acceptors achieved impressive PCEs exceeding 16%. This highlights the significant potential of the novel molecular design strategy, which utilizes an A-D-A-type configuration featuring a linear-shaped molecular backbone. We further incorporated partially fluorinated side chains into the heteroheptacene donor core to enhance the fill factor (FF) [51]. Additionally, we employed a dimerization strategy to increase the photocurrent values and stability of PSCs based on the M-series acceptors, resulting in PSCs with PCEs up to 17.2% [52]. The removal of *sp*^3^-hybridized carbon atoms from the heteroheptacene core endows these M-series acceptors with relatively planar conjugated backbones, in contrast to Y-series acceptors, which possess longer and more twisted conjugated backbones. Consequently, M-series acceptors are expected to have advantages in forming stable phase morphologies with ordered intermolecular packing and reduced diffusion coefficients. However, it remains challenging to leverage the more planar conjugated backbones of M-series acceptors to achieve PSCs with both good stability and high efficiencies comparable to or exceeding those of Y-series acceptors.

**Scheme 1.**
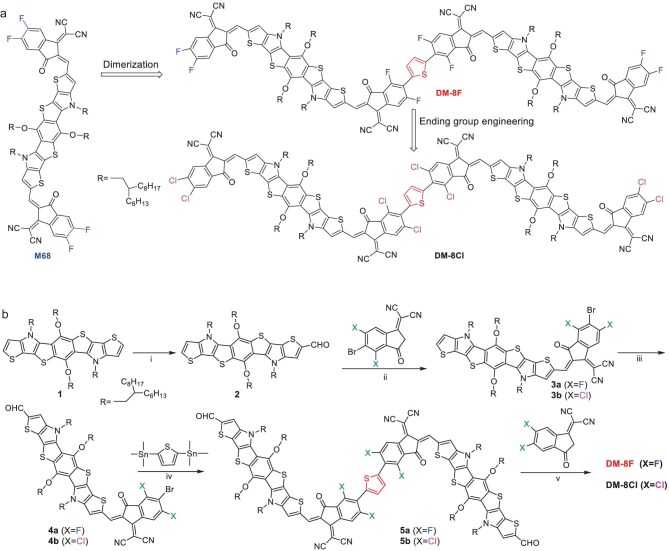
(a) Molecular structures of M68, DM-8F, and DM-8Cl. (b) Synthetic route for the dimerized acceptors DM-8F and DM-8Cl. Reagents and conditions: (i) POCl_3_/DMF, 83%; (ii) pyridine, 73% for **3a** and 83% for **3b**; (iii) POCl_3_/DMF, 89% for **4a** and 81% for **4b**; (iv) Pd_2_(dba)_3_/P(o-tolyl)_3_, 65% for **5a** and 72% for **5b**; (v) BF_3_·OEt_2_/Ac_2_O, 88% for **DM-8F** and 85% for **DM-8Cl**.

In this study, we designed and synthesized two dimerized M-series acceptors (DM-8F and DM-8Cl, shown in Scheme 1a) featuring an A-D-A-type configuration. For comparison, we also prepared the corresponding monomeric acceptor (M68 in Scheme 1a), which possesses the same side-chains and terminal groups as DM-8F. The structural differences between DM-8F and DM-8Cl arise from the varying halogen atoms in their terminal groups, which influence the planarity of their backbones. We conducted a systematic investigation into how molecular structure influences various properties, including optical absorption, dipole moments, dielectric constants, blend film morphology, charge transport characteristics, exciton diffusion length, photovoltaic efficiency, and both photo- and thermal stability of PSCs. When blended with the wide bandgap polymer PM6, the DM-8F–based PSC exhibited an outstanding PCE of 19.39% (certified at 19.20%), with an open-circuit voltage (*V*_OC_) of 0.881 V, a short-circuit current density (*J*_SC_) of 28.0 mA/cm^2^, and an FF of 0.786. To the best of our knowledge, the certified PCE of 19.20% is the highest reported among all A-D-A-type NFAs. Additionally, an impressive PCE of 15.72% was achieved for minimodule devices with an effective area of 11.09 cm^2^ using DM-8F as the acceptor material. Furthermore, the DM-8F–based PSCs exhibited excellent photo- and thermal stability due to the higher glass transition temperature (*T*_g_) and lower diffusion coefficient of DM-8F.

## RESULTS AND DISCUSSION

The synthetic routes for DM-8F and DM-8Cl are illustrated in Scheme 1b, while the synthesis of the monomeric acceptor M68 is described in the Supplementary Notes. As shown in the scheme, we employed a mono Vilsmeier–Haack reaction to functionalize the electron-rich heteroheptacene donor core (**1**) with a single aldehyde group. A 1-fold Knoevenagel condensation reaction between Compound **2** and 2-(5-bromo-4,6-difluoro-3-oxo-2,3-dihydro-1H-inden-1-ylidene)malononitrile (or 2-(5-bromo-4,6-dichloro-3-oxo-2,3-dihydro-1H-inden-1-ylidene) malononitrile) resulted in the formation of Compounds **3a-b**, which were subsequently subjected to another 1-fold Vilsmeier–Haack reaction. Following this, a 2-fold Stille coupling reaction between Compounds **4a-b** and 2,5-bis(trimethylstannyl)thiophene yielded Compounds **5a-b**. The final dimerized acceptors (DM-8F and DM-8Cl) were obtained through a 2-fold Knoevenagel condensation reaction between Compounds **5a-b** and 2-(5,6-difluoro-3-oxo-2,3-dihydro-1H-inden-1-ylidene)malononitrile (or 2-(5,6-dichloro-3-oxo-2,3-dihydro-1H-inden-1-ylidene)malononitrile). The chemical structures of DM-8F and DM-8Cl were confirmed by nuclear magnetic resonance (NMR) spectroscopy and high-resolution mass spectrometry.

**Figure 1.**
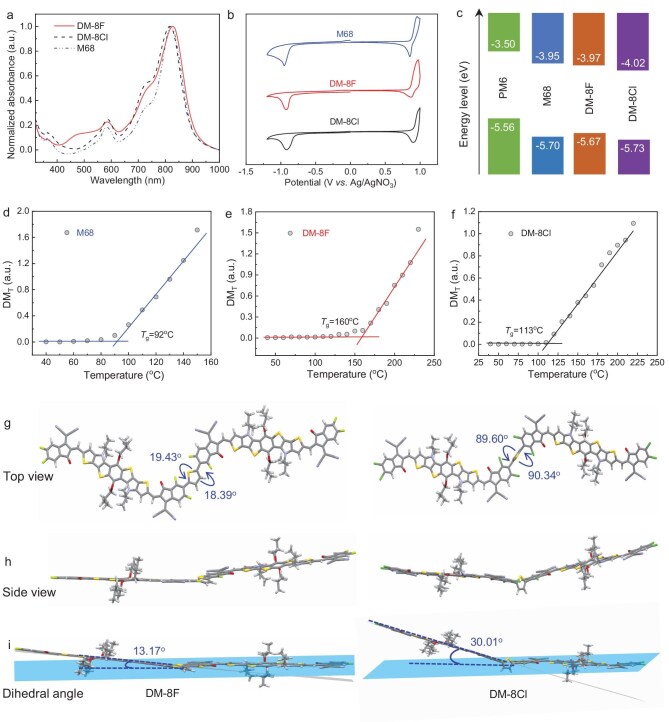
(a) Normalized absorption spectra of M68, DM-8F, and DM-8Cl in thin films. (b) Cyclic voltammograms of M68, DM-8F, and DM-8Cl. (c) Energy level diagram of M68, DM-8F, and DM-8Cl. (d–f) Plots of the absorbance deviation metric for M68 (d), DM-8F (e), and DM-8Cl (f) films as a function of annealing temperature. (g–i) Top views (g), side views (h), and dihedral angles (i) of optimized geometries of the dimerized acceptors obtained by DFT calculations.

The optical properties of DM-8F, DM-8Cl, and M68 in thin films and chloroform solutions were studied using UV–vis-NIR absorption spectroscopy (Fig. 1a and Fig. S3d). Notably, both DM-8F and DM-8Cl exhibit broader absorption bands, with full width at half maximum (FWHM) values of 137 nm and 118 nm, respectively, compared to M68 which has a smaller FWHM value of 93 nm. The broader absorption of DM-8F was also observed in thin film which is attributed to its extended conjugation length and increased molecular interaction modes compared to M68. In the two DAs, DM-8F displays a red-shifted absorption compared to DM-8Cl. Notably, DM-8F exhibits stronger absorption in the 400–550 nm range, which can be attributed to the shorter effective π-conjugation and weaker intramolecular charge transfer (ICT) effect in DM-8Cl [9]. Detailed optical parameters are provided in Table S1. The energy levels of the lowest unoccupied molecular orbital (LUMO) and the highest occupied molecular orbital (HOMO) for the three acceptors were estimated based on cyclic voltammetry (CV) (Supplementary Notes) and the resulting CV curves are depicted in Fig. 1b. From the monomeric M68 to the dimerized acceptor DM-8F, the *E*_HOMO_ slightly increased from −5.70 to −5.67 eV, while the *E*_LUMO_ slightly decreased from −3.95 to −3.97 eV. This change resulted in a marginally reduced bandgap for DM-8F, consistent with the optical absorption results. From DM-8F to DM-8Cl, both *E*_HOMO_ and *E*_LUMO_ decreased, from −5.67 to −5.73 eV and from −3.97 to −4.02 eV, respectively, indicating differing effects of halogenation between the two DAs. The energy diagram for the three acceptors and the polymer donor PM6, is depicted in Fig. 1c. The electronic properties and optimized geometries of the three acceptors were further investigated using density functional theory (DFT) calculations. To simplify computations, the 2-hexyldecyl chains were replaced with isobutyl groups. The calculated HOMO energy levels of M68, DM-8F, and DM-8Cl are −5.72, −5.69, and −5.74 eV, respectively, while the LUMO energy levels are −3.68, −3.78, and −3.76 eV. The energy level variations from the DFT calculations generally align with the CV results. The distributions of electrostatic surface potentials (ESPs) for the DAs (DM-8F and DM-8Cl) are quite similar to that of the monomeric M68 (Fig. S1). For all three acceptors, negative ESPs are primarily concentrated on strongly electronegative units, such as fluorine, chlorine, cyano, and carbonyl groups. In contrast, positive ESPs are located on the conjugated surfaces and alkyl chains. The ESP distribution reflects the uneven charge density, which, in turn, influences intramolecular electrostatic interactions.

All three acceptors exhibit excellent thermal stability, with decomposition temperatures (*T*_d_) exceeding 300°C (Table S1 and Fig. S2a). Due to the amorphous and disordered nature of the two DAs and M68, they do not exhibit any distinct transition peaks in the differential scanning calorimetry (DSC) measurements (Fig. S2b). The *T*_g_ is a crucial parameter for predicting morphological stability. The *T*_g_ values of acceptor materials are associated with their diffusion and crystallization processes in the active layer, and a higher *T*_g_ value is believed to promote the formation of a stable microstructural morphology. In this study, the *T*_g_ values of the acceptors were estimated by quantifying the deviation metric based on the change in absorbance of each film during continuous thermal annealing (Fig. S3), following the method reported by Ade *et al.* [53]. Figure 1d–f shows the deviation metric plotted against the annealing temperature for the three acceptor films. Through linear fitting, the *T*_g_ values (from the optical deviation metric) of M68, DM-8F, and DM-8Cl were estimated to be 92, 160, and 113°C, in that order. A notable increase in *T*_g_ is observed from M68 to the dimerized acceptor DM-8F. However, when the fluorine atoms in the ending groups are replaced by chlorine atoms, a significant decrease in *T*_g_ is noted, likely related to their geometric differences. From the top views of the molecular geometries (Fig. 1g–i), it can be observed that the connecting thiophene ring in DM-8Cl is nearly perpendicular to the benzene ring in the indanone units, with dihedral angles of ∼90°. In contrast, the connecting thiophene ring in DM-8F is coplanar with the benzene ring in the indanone units, exhibiting dihedral angles of ∼19°. This difference can be attributed to the relatively larger size of chlorine atoms compared to fluorine atoms. From the side views, it can be observed that the dihedral angles between two neighboring electron-withdrawing groups (the central indanone groups) of DM-8F are theoretically calculated to be ∼13°, which is significantly smaller than the 38.74° observed in the counterpart based on the Y6 derivative [36]. However, when the fluorine atoms are replaced with chlorine atoms, the corresponding dihedral angles increase significantly to 30.01° (Fig. 1i). It has been shown that there is a significant correlation between the inferred diffusion coefficients at 85^o^C (*D*_85_) of acceptors blended with specific donor materials and their *T*_g_ values. According to the method proposed by Ade *et al.* (Supplementary Notes), *D*_85_ decreases exponentially with increasing *T*_g_ value [53]. Thus, *D*_85_ values of DM-8F, DM-8Cl, and M68 were determined to be 9.3 × 10^−22^, 1.2 × 10^−18^, and 1.5 × 10^−17^ cm^2^ s^−1^, respectively. The markedly reduced *D*_85_ value of DM-8F may contribute to improved thermal stability of the resulting solar cells, as discussed in the following section. Furthermore, DFT calculations revealed that the dipole moments of M68, DM-8F, and DM-8Cl are 0.00, 1.11, and 1.04 Debye, respectively (Fig. S1d). Owing to its symmetrical structure, M68 exhibits no dipole moment. In contrast, the dimerized acceptors show significantly enhanced dipole moments, which are advantageous for achieving higher dielectric constants.

**Figure 2.**
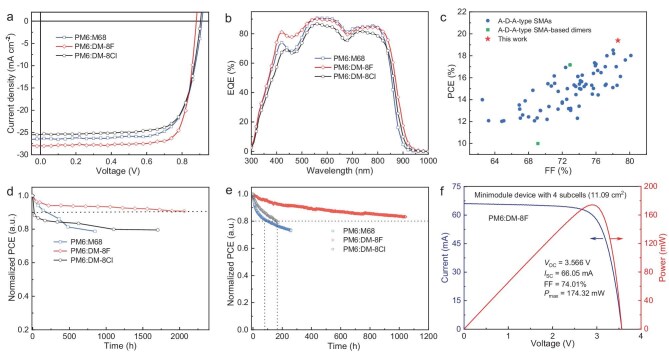
(a) *J-V* curves of best-performing PSCs based on the PM6:M68, PM6:DM-8F, and PM6:DM-8Cl blends. (b) EQE spectra of the best-performing PSCs. (c) Comparison of device parameters of A-D-A-type acceptor-based OSCs between this work and the previously reported systems (the original data are provided in Table S9). (d) Normalized PCEs of the PSCs based on PM6:M68, PM6:DM-8F, and PM6:DM-8Cl under thermal annealing at 80°C for different times. (e) MPP stability tests of the unencapsulated PSCs based on PM6:M68, PM6:DM-8F, and PM6:DM-8Cl. (f) *I-V* and power curves of the minimodule based on PM6:DM-8F.

To evaluate the photovoltaic performance of the three NFAs, we fabricated conventional PSCs using PM6 as the donor material. The PSCs were optimized under various conditions (Tables S2–S8). Detailed device fabrication procedures are provided in the Supplementary Notes. Following optimization of the solvent, solvent additives, and thermal annealing conditions, the performance of the layer-by-layer (LBL) processed devices was further enhanced by using the DM-8F acceptor at various concentrations to optimize the thickness of the active layer (Table S5). Figure 2a and b illustrates the current density *versus* voltage (*J–V*) curves and the EQE spectra, respectively, while Table 1 and Fig. S4 present the corresponding device parameters. The best-performing PM6:M68-based device showed a PCE of 17.92%, with a *J*_SC_ of 26.6 mA cm^−2^, a *V*_OC_ of 0.914 V, and an FF of 0.737. The best-performing PM6:DM-8Cl–based device delivered a PCE of 17.02%, with a *V*_OC_ of 0.903 V, a *J*_SC_ of 25.4 mA cm^−2^, and an FF of 0.742. In contrast, the best-performing PM6:DM-8F–based device exhibited a PCE of 19.39%, with a *J*_SC_ of 28.0 mA cm^−2^, a *V*_OC_ of 0.881 V, and an FF of 0.786. Notably, the PM6: DM-8F–based device displayed a decreased *V*_OC_ value, which can be attributed to the deeper LUMO energy level of DM-8F. The PM6:DM-8F–based device showed an increased *J*_SC_ value in comparison with the PM6:M68-based device primarily due to the increased EQE values in the 400–500 nm range. This improvement in current density results partly from the stronger intrinsic absorption of the DM-8F film in this wavelength range (Fig. 1a) and partly from the improved blend film morphology, which further increases the absorption intensity (Fig. S5a). The best-performing PM6:DM-8F–based device was assessed by the National Photovoltaic Product Quality Inspection and Testing Center of China, where a certified PCE of 19.20% was achieved, as illustrated in Fig. S6. Importantly, the certified PCE of 19.20% is the highest reported to date for single-junction PSCs utilizing A-D-A-type acceptors (Fig. 2c and Table S9), and it is also higher than those of many Y6-derived dimerized acceptors (Table S10 and Fig. S7). In addition, a series-connected four-subcell minimodule device (schematic and photograph shown in Fig. S8a and S8b) with an active area of 11.09 cm^2^ was successfully fabricated employing the PM6:DM-8F active layer. Figure 2f depicts the measured *I-V* curve and output power of the minimodule device, along with the corresponding photovoltaic parameters. The minimodule achieved a PCE of 15.72% with a *V*_OC_ of 3.566 V, an *I*_SC_ of 66.05 mA, and an FF of 0.740.

**Table 1.** Device parameters of PSCs based on the acceptors under AM 1.5 G, 100 mW/cm^2^.

Active layer	PCE (%)^a^	FF	*V*_OC_ (V)	*J*_SC_ (mA cm^−2^)	*J*_SC_^cal^ (mA cm^−2^) ^b^
PM6:M68	17.92 (17.54 ± 0.25)	0.737	0.914	26.6	25.5
PM6:DM-8Cl	17.02 (16.60 ± 0.20)	0.742	0.903	25.4	24.5
PM6:DM-8F	19.39 (19.09 ± 0.15)	0.786	0.881	28.0	26.7
PM6:DM-8F (Certified)^c^	19.20	0.794	0.874	27.7	

a Figures in parentheses are average and standard deviation data obtained from 20 devices. ^b^The integrated current densities from the EQE spectra are provided in Fig. S8c. ^c^The certified PCE from an independent third-party laboratory.

The thermal stability of PSCs was further investigated by continuously heating the devices at 80^o^C in an inert atmosphere. In this study, we fabricated inverted devices with the structure of indium tin oxide (ITO)/ZnO/active layer/MoO_3_/Ag for thermal stability testing, aiming to mitigate the negative effects of organic interlayers on device stability. The fabrication and optimization details of the inverted devices are presented in the Supplementary Notes and Table S8. The dependence of PCEs on annealing time for the three devices is illustrated in Fig. 2d. After thermal annealing at 80°C for 2060 hours, the efficiency of PM6:DM-8F–based PSC remained at 90.7% of its initial value. For the device based on the PM6:DM-8Cl blend, 79.5% of its initial PCE was maintained after annealing at 80°C for 1704 hours. In contrast, the PM6:M68-based PSC only maintained 78.8% of its initial PCE after being annealed at 80°C for 848 hours. Based on this data, their *T*_90_ lifetimes (the duration of time it takes for the PCE to degrade to 90% of its initial value) can be estimated. As shown in Fig. 2d, the PM6:DM-8F–based device offers a *T*_90_ lifetime of >2060 hours, which is around 13 times greater than that of the PM6:M68-based device (*T*_90_ = 150 hours). The greatly improved thermal stability of the PM6:DM-8F–based PSC is primarily related to the higher *T*_g_ value of 160°C for DM-8F compared to the other two PSCs based on PM6:DM-8Cl and PM6:M68. The exceptional thermal stability of DM-8F–based devices makes them highly competitive for future practical applications. At the same time, the light stability of the PSCs based on the three acceptors was investigated. As shown in Fig. 2e, after continuous one-sun-equivalent illumination for 1044 hours, the PM6:DM-8F–based device retained 83.2% of its initial PCE indicating a *T*_80_ lifetime of >1044 hours. In contrast, the PM6:DM-8Cl–based and the PM6:M68-based devices exhibited relatively shorter *T*_80_ lifetimes of 163 hours and 103 hours, respectively. These findings reveal that employing dimerized M-series acceptors characterized by an elevated *T*_g_ in PSC fabrication leads to both enhanced thermal- and photostability in the resulting devices. It should be noted that the PM6:DM-8F–based device showed comparable or superior thermal stability under continuous heating at 80°C compared to the counterparts based on Y6-derived dimerized acceptors [33–36].

We carried out a comprehensive analysis of energy loss (*E*_loss_) in the three PSCs by obtaining their electroluminescence (EL) spectra and Fourier-transform photocurrent spectroscopy external quantum efficiency (FTPS-EQE) (Supplementary Notes). Generally, the *E*_loss_ in PSCs can be categorized into three components, expressed by the following equation: *E*_loss_=Δ*E*_1_+Δ*E*_2_+Δ*E*_3_=(*E*_g_-$qV_{{\mathrm{oc}}}^{{\mathrm{SQ}}}$)+($qV_{{\mathrm{oc}}}^{{\mathrm{SQ}}} - qV_{{\mathrm{oc}}}^{{\mathrm{rad}}}$)+($qV_{{\mathrm{oc}}}^{{\mathrm{rad}}} - q{V}_{{\mathrm{oc}}}$), where $qV_{{\mathrm{oc}}}^{{\mathrm{SQ}}}$ is the possible *V*_OC_ under the Shockley–Queisser limit, and $qV_{{\mathrm{oc}}}^{{\mathrm{rad}}}$ is the *V*_OC_ only considering the radiative recombination (Supplementary Notes). Δ*E*_1_ results from the radiative energy loss exceeding the bandgap, an occurrence that is inevitable for all solar cells. In this study, the Δ*E*_1_ values for PM6:DM-8F, PM6:DM-8Cl, and PM6:M68 are 0.262, 0.263, and 0.264 eV, respectively (Fig. S9a). Δ*E*_2_ represents the radiative energy loss occurring below the bandgap and is correlated with the energy difference (Δ*E*_CT_) between the bandgap (*E*_g_^PV^) and the charge-transfer state (*E*_CT_) of the PSCs. For the PM6:DM-8F–, PM6:DM-8Cl–, and PM6:M68-based devices, the *E*_g_^PV^ values were determined to be 1.446, 1.456, and 1.472 eV, in that order, by measuring the emission and absorption spectra of the corresponding blend films (Fig. S5). By fitting the FTPS-EQE and EL spectra, the *E*_CT_ values for PM6:DM-8F–, PM6:DM-8Cl–, and PM6:M68-based devices were determined to be 1.411, 1.426, and 1.450 eV, in that order (Fig. S9d–S9f). Consequently, the Δ*E*_CT_ values for the PM6:DM-8F–, PM6:DM-8Cl–, and PM6:M68-based devices are 0.035, 0.030, and 0.022 eV, in that order. As illustrated in Fig. S9a, the observed variations in Δ*E*_CT_ values show a direct correlation with the corresponding Δ*E*_2_ values. The PM6:DM-8F–, PM6:DM-8Cl–, and PM6:M68-based devices exhibit progressively lower Δ*E*_2_ values of 0.050, 0.041, and 0.038 eV, respectively. As a non-radiative recombination loss, Δ*E*_3_ can be quantified as (-*k*T/*q*)ln(EQE_EL_), where T is the Kelvin temperature, *k* is the Boltzmann constant, and EQE_EL_ is the electroluminescence quantum efficiency of the PSC. The reduction of Δ*E*_CT_ in PSCs is widely recognized to promote electronic coupling between CT and localized excitation (LE) states. This synergistic interaction allows the CT state to harness the strong luminescent characteristics of the LE state, thereby enhancing its emission efficiency [54]. Furthermore, diminished Δ*E*_CT_ values facilitate the reverse transition from the CT to LE state, creating an energy transfer pathway that enables radiative recombination of the CT state through the emissive LE state. This photophysical process effectively mitigates non-radiative recombination losses (quantified as Δ*E*_3_) in PSCs. To experimentally verify this mechanism and establish quantitative correlations with Δ*E*_3_, we conducted external quantum electroluminescence EQE_EL_ measurements across three PSCs (Supplementary Notes). As shown in Fig. S9b, the PM6:DM-8F–based device exhibited an EQE_EL_ of 1.43 × 10^−4^, corresponding to a Δ*E*_3_ of 0.229 eV. In contrast, the PM6:DM-8Cl– and PM6:M68-based devices showed higher EQE_EL_ values of 1.59 × 10^−4^ and 1.69 × 10^−4^, leading to lower Δ*E*_3_ values of 0.226 and 0.224 eV, respectively. These variations in Δ*E*_3_ are consistent with the PLQY trends observed for the three acceptor materials (Table S1). Based on this data, the *E*_loss_ of the PM6:DM-8F–based device was calculated to be 0.541 eV, which is slightly higher than the values of 0.530 and 0.526 eV observed for the PM6:DM-8Cl– and PM6:M68-based devices (Table S11). These findings demonstrate that the enhanced *V*_OC_ values in the PM6:DM-8Cl– and PM6:M68-based devices arise from the upshifted *E*_CT_ caused by the higher LUMO energy levels of DM-8Cl and M68, which simultaneously suppress radiative and non-radiative recombination processes.

It has been established that a higher dielectric constant (*ε*_r_) of an organic photovoltaic material typically results in a lower exciton binding energy (*E*_b_) [54]. In this study, we employed impedance spectroscopy with parallel-plate capacitance devices to determine the dielectric constants of the three acceptors and the corresponding blend films (Supplementary Notes). The frequency dependence of the dielectric constants for DM-8F, DM-8Cl, and M68, within the range of 20 to 2 × 10^5^ Hz, is illustrated in Fig. S10a. In the wide frequency range, the *ε*_r_ values for DM-8F and DM-8Cl are significantly higher than that of M68. For example, at the frequency of 20 Hz, the *ε*_r_ values for DM-8F and DM-8Cl are 5.02 and 4.81, respectively, which are both higher than the *ε*_r_ value of 3.42 for M68. The increased *ε*_r_ values of both DAs (DM-8F and DM-8Cl) can be attributed to their larger dipole moments compared to M68, as we previously discussed (Fig. S1d). In addition, when both DAs were blended with PM6, the resulting blend films of PM6:DM-8F and PM6:DM-8Cl still exhibit higher *ε*_r_ values compared to PM6:M68 (Fig. S10b).

The energy barrier or activation energy (*E*_a_) that governs exciton dissociation into free charge carriers is a critical parameter in the characterization of optoelectronic materials. *E*_a_ can be quantitatively determined through temperature-dependent photoluminescence analysis (Supplementary Notes) using the modified Arrhenius relationship: *I*(T) = *I*_0_/[1 + Aexp(−*E*_a_/*k*_B_*T*)], where *I*_0_ denotes the extrapolated emission intensity at absolute zero, *T* is the measurement temperature, and *k*_B_ represents the Boltzmann constant [54]. To systematically investigate the correlation between the dielectric constant and exciton dissociation dynamics, we employed temperature-modulated photoluminescence spectroscopy to extract *E*_a_ values across three acceptors with varying dielectric constants. As illustrated in Figs S11–S13, the total photoluminescence (PL) intensity for the three samples varied as a function of temperature, ranging from 80 to 300 K. For all three samples, the PL intensity decreased with increasing temperature, suggesting that the photogenerated excitons tend to dissociate into free charge carriers at higher temperatures. The estimated *E*_a_ values for DM-8F and DM-8Cl are 24.51 meV and 24.42 meV (Fig. S9c), respectively, both of which are significantly lower than that for M68 (31.65 meV). The reduced *E*_a_ values for the DAs indicate lower exciton dissociation barriers, which favorably facilitate exciton dissociation and the charge transfer process. Considering that *E*_b_ represents the energy difference between the charge-separated (CS) state and the first singlet excited state (S_1_), the reduced *E*_a_ values for the DAs indicate a corresponding decrease in their *E*_b_ values [55]. Thus, our findings suggest that DAs offer the advantage of a higher dielectric constant, which subsequently reduces exciton binding energy. This reduction is beneficial for enhancing charge currents in the resulting photovoltaic devices. We then fabricated photovoltaic devices using active layers composed solely of electron acceptor materials to evaluate the exciton dissociation capabilities of DM-8F, DM-8Cl, and M68 films. The external quantum efficiency (EQE) spectra and the corresponding integrated current density curves of the photovoltaic devices based on DM-8F, DM-8Cl, and M68 are presented in Fig. S14. Both the DM-8F– and DM-8Cl–based devices demonstrate an enhanced photoresponse across the absorption range of 300 to 900 nm compared to the M68-based device. Additionally, the current densities obtained by integrating the EQE curves with the AM 1.5 G photon flux were 0.46 mA cm^−2^ and 0.47 mA cm^−2^ for the devices based on DM-8F and DM-8Cl, respectively. These values are significantly larger than the current density for the M68-based device (0.19 mA cm^−2^). The improved current densities and EQEs of the DM-8F– and DM-8Cl–based devices, in comparison to the M68-based device, can be attributed to the lower binding energy resulting from their higher dielectric constants.

To gain a deeper understanding of the highest *J*_SC_ value in the PM6:DM-8F–based device, we conducted measurements of the photocurrent density (*J*_ph_) as a function of effective voltage (*V*_eff_) (Supplementary Notes). This analysis enabled us to evaluate the charge dissociation process, from which we determined the exciton dissociation efficiency (*P*_diss_). As shown in Fig. S15, the *P*_diss_ of the PM6:DM-8F device is 98.5%, which is higher than that of PM6:M68 (98.1%) and PM6:DM-8Cl (98.1%). The elevated *P*_diss_ value suggests a more efficient charge dissociation in the PM6:DM-8F–based device. The dependencies of *J*_sc_ values on light intensity (*P*_light_) were studied to reveal the charge recombination behavior within the devices based on the three acceptors. The equation *J*_sc_ ∝ (*P*_light_)^α^ can be used to express the relationship between *J*_SC_ and *P*_light_, where the exponential factor α indicates the extent of bimolecular recombination. By fitting the corresponding curves, α values of 0.992, 0.978, and 0.974 were obtained for the PM6:DM-8F–, PM6:DM-8Cl–, and PM6:M68-based devices, respectively (Fig. S16). The highest α value for the PM6:DM-8F–based device suggests that charge recombination is most efficiently suppressed in the device, which accounts for its highest FF and *J*_sc_ values.

To assess the performance variation of PSCs based on three different acceptor materials, the space charge limited current (SCLC) method was used to determine their charge transport properties (Supplementary Notes). As shown in Fig. S17, the calculated electron mobilities (*μ*_e_) for PM6:DM-8F, PM6:DM-8Cl, and PM6:M68 blend films are 3.73 × 10^−4^, 3.33 × 10^−4^, and 1.40 × 10^−4^ cm^2^ V^−1^ s^−1^, in that order. And the hole mobilities (*μ*_h_) for PM6:DM-8F, PM6:DM-8Cl, and PM6:M68 are 3.63 × 10^−4^, 2.71 × 10^−4^, and 2.94 × 10^−4^ cm^2^ V^−1^ s^−1^, in that order (Table S12). This data suggests that both DAs exhibit an increased electron mobility compared to M68. Owing to the highest carrier mobilities and well-balanced electron-hole transport characteristics in the PM6:DM-8F blend, the resulting PSCs demonstrate superior performance with enhanced FF and *J*_SC_ values.

**Figure 3.**
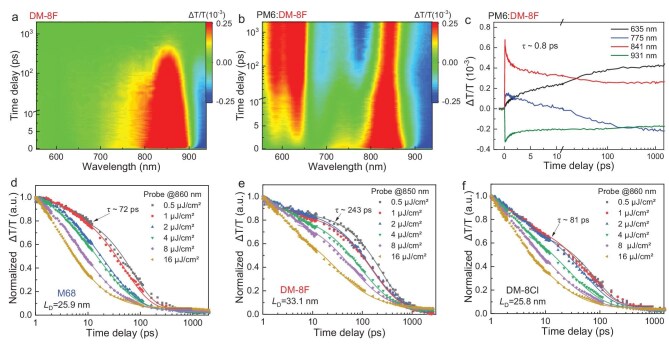
(a and b) 2D TAS images of DM-8F and PM6:DM-8F. (c) TA spectra of PM6:DM-8F at different time delays. (d–f) Decay dynamics of excitons in M68 (d), DM-8F (e), and DM-8Cl (f) films with varying pump fluences.

The charge-transfer dynamics were further investigated using femtosecond transient absorption (TA) spectroscopy (Supplementary Notes), as illustrated in Fig. 3a–c. Figure 3a and b compares the TA data recorded from a neat film of DM-8F and a blend film of PM6:DM-8F by selective excitation of the acceptor using 860 nm laser pulses. In the neat film of DM-8F, the TA kinetics are dominated by the decay of the acceptor’s ground-state bleach (GSB), which has a lifetime of ∼243 ps within its resonant band. However, in the blend film, the acceptor’s GSB signal decreases rapidly, coinciding with the emergence of the donor’s GSB signal at ∼630 nm. This observation suggests an ongoing hole transfer process from the acceptor (DM-8F) to the donor (PM6). Subsequently, a pronounced excited-state absorption (ESA) feature at ∼760 nm, associated with free carriers, emerges at longer timescales in the blend. Notably, the blend sample exhibits an ultrafast decay component of the acceptor GSB (∼0.8 ps) at the earliest time scale (Fig. 3c), which can be attributed to rapid hole transfer across the donor-acceptor interface. Given the long-lived nature of the acceptor species, the near-unity efficiency of hole transfer suggests that most excitons at the interface successfully contribute to charge generation. Such efficient interfacial charge transfer dynamics are also observed in the blends incorporating the other two acceptors (M68 and DM-8Cl in Fig. S18), highlighting the general applicability of this mechanism.

Although the interfacial charge separation efficiencies are comparable across all three blends, the resulting free charge signals exhibit distinct characteristics (Fig. S18). This discrepancy is likely attributed to differences in bulk exciton dynamics, which was investigated by using fluence-dependent TA spectroscopy. Following established literature methods [56,57], exciton diffusion lengths (*L*_D_) are determined through exciton-exciton annihilation (EEA) analysis (Supplementary Notes). Figure 3d-f depicts the decay dynamics of the excitons in the DM-8F, DM-8Cl, and M68 films, in that order. Bimolecular exciton annihilation rate constants (*α*) and intrinsic exciton decay rate constants (*κ*) were obtained by globally fitting the decay dynamics of the three acceptors. Based on the *α* and *κ* values, the corresponding *L*_D_ values were calculated, and the detailed calculation processes, along with related parameters, are provided in Table S13. Notably, DM-8F exhibits a significantly longer *L*_D_ of 33.1 nm compared to M68 (25.9 nm) and DM-8Cl (25.8 nm), indicating the enhanced exciton transport capability of the DM-8F films. This finding is consistent with the exceptional exciton lifetime of DM-8F (243 ps), which is considerably longer than those of the other two acceptors (72 ps for M68 and 81 ps for DM-8Cl). The extended conjugation length and more planar molecular backbone in DM-8F could enhance molecular interactions and potentially slow down recombination pathways thereby extending the exciton diffusion range. This increased transport range promotes more efficient charge collection at the donor-acceptor interface, ultimately improving device performance.

**Figure 4.**
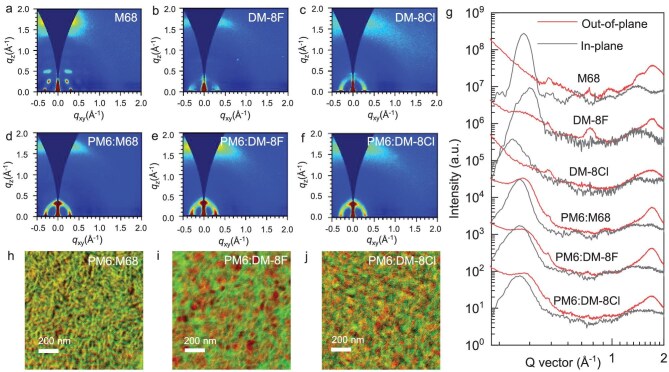
2D GIWAXS patterns (a–f) and 1D line-cut profiles (g) of the neat acceptors and blend films. (h–j) Combined PiFM images at the wavenumbers of 1540 cm^−1^ (green representing acceptor) and 1650 cm^−1^ (red representing PM6).

Grazing-incidence wide-angle X-ray scattering (GIWAXS) measurements were conducted to investigate the π–π-packing and molecular orientation of the acceptor materials in both pristine and blended films (Supplementary Notes). Figure 4a–g depicts the 2D GIWAXS patterns as well as the corresponding 1D line-cut profiles in the in-plane (IP) and out-of-plane (OOP) directions. All three acceptor materials showed a face-on orientation relative to the substrate, as evidenced by the intense lamellar (100) peak in the IP direction and a distinct (010) peak corresponding to π–π stacking in the OOP direction. However, both the pristine DM-8F film and the PM6:DM-8F blend film showed a more compact π–π-packing with the smallest packing distances of 3.62 and 3.68 Å, respectively. In contrast, the pristine DM-8Cl film and the PM6:DM-8Cl blend film exhibited π–π-packing distances of 3.77 and 3.73 Å. Additionally, a π–π-packing distance of 3.69 Å was observed for both the pristine M68 film and the PM6:M68 blend film. In going from the small-molecule acceptor (M68) to the DAs (DM-8Cl or DM-8F), a reduced crystal correlation length (CCL) was observed indicating the relatively high crystallinity of the small-molecule acceptor (Table S14). Among the three acceptors, DM-8F showed the shortest π–π-packing distance in its pure film as well as its blend film which can be attributed the extended and more planar conjugation backbone of DM-8F. We further used the GIWAXS measurement to learn the morphology stability of the blend films of PM6:DM-8F and PM6:M68 before and after thermal annealing at 100°C. As shown in Fig. S19, 2D GIWAXS patterns indicated that after thermal aging at 100°C for 72 hours, more edge-on orientations can be found for the PM6:M68 blend which is harmful to device performance. However, 2D GIWAXS patterns of PM6:DM-8F blend film barely change under the same thermal annealing conditions. The results suggest that the morphology of PM6:DM-8F is more thermally stable at elevated temperatures, explaining the enhanced thermal stability of devices based on PM6:DM-8F, in comparison with those based on PM6:M68.

The surface morphology of blend films based on PM6:DM-8F, PM6:DM-8Cl, and PM6:M68 was assessed using atomic force microscopy (AFM) as illustrated in Fig. S20. Fiber-like morphology is evident in all three blend films. However, the PM6:DM-8F and PM6:M68 blend films exhibited lower roughness (*R*_q_) values (1.18 nm for PM6:DM-8F and 1.14 nm for PM6:M68) compared to the PM6:DM-8Cl blend (1.42 nm), which can be attributed to the more planar molecular structures of the former two. The reduced roughness indicates a more uniform surface, which is beneficial for forming an ohmic contact with decreased series resistance. Furthermore, photo-induced force microscopy (PiFM), which employs localized near-field infrared imaging to identify the chemical properties associated with thin-film morphology, was utilized to assess this morphology (Supplementary Notes). According to the characteristic peaks of the acceptors (DM-8F, DM-8Cl, and M68) and the donor (PM6), the absorption wavenumbers of 1540 cm^−1^ and 1650 cm^−1^ were used to distinguish the acceptor and donor phases in the blend films [26,58]. The corresponding PiFM phase images of the PM6:DM-8F, PM6:DM-8Cl, and PM6:M68 blends are presented in Fig. S21. Again, fibril network structures can be observed in all three blend films. The overlay of the two images (Fig. 4h–j) reveals clear phase separation, with the acceptor and donor domains in close contact. Figure S22 depicts line profiles across the PiFM images, and the FWHM value of the peaks was utilized to estimate the diameter of the fibrils. An average diameter of 14.9 nm was found for the PM6:DM-8F blend, which is slightly smaller than those for the PM6:DM-8Cl blend (17.1 nm) and the PM6:M68 blend (18.3 nm). The fibril-like morphology with smaller fibril sizes in the PM6:DM-8F blend is consistent with the high FF and *J*_SC_ values observed in the corresponding device.

## CONCLUSION

We have designed and synthesized two dimerized acceptors, DM-8F and DM-8Cl, along with their corresponding A-D-A-type small-molecule counterpart M68. Through comprehensive characterizations, we systematically elucidated the structure-property relationships of these acceptors, focusing on their optical absorption bandgaps, energy level alignments, dielectric constants, glass transition temperatures, charge carrier mobilities, exciton diffusion lengths, and ultimately, photovoltaic performance. The dimerized acceptors exhibit broader absorption spectra, higher *T*_g_ values, and greater dielectric constants than the small-molecule reference M68. Theoretical calculations reveal that in DM-8Cl, the chlorinated end group forms a larger dihedral angle with the central thiophene ring, resulting in a less planar dimerized structure compared to its fluorinated counterpart, DM-8F. This structural distortion results in a lower *T*_g_ and a larger π–π stacking distance in DM-8Cl. PSCs were fabricated by blending each acceptor with the polymer donor PM6. The best-performing DM-8F–based device achieved a PCE of 19.39%, significantly outperforming the devices based on DM-8Cl (PCE = 17.02%) and M68 (PCE = 17.92%). Notably, the best-performing DM-8F–based device attained a third-party certified PCE of 19.20%, which, to the best of our knowledge, represents the highest reported value to date among all A-D-A-type acceptor materials. A minimodule device based on PM6:DM-8F blend with an effective area of 11.09 cm^2^ also delivered an outstanding PCE of 15.72%. Furthermore, the PM6:DM-8F–based PSC achieved a long *T*_90_ lifetime exceeding 2060 hours under continuous heating at 80^o^C, which is around 13 times greater than those of the PM6:M68- and PM6:DM-8Cl–based devices (both <150 hours). This markedly improved thermal stability of the PM6:DM-8F–based PSC is attributed to the higher *T*_g_ of DM-8F compared to the other two acceptors. The PM6:DM-8F–based device also exhibited improved photostability, with a *T*_80_ lifetime exceeding 1000 hours, far in excess of the PM6:DM-8Cl– and PM6:M68-based devices (<200 hours). Our findings reveal that the dimerized acceptor DM-8F, derived from the planar M-series A-D-A-type acceptor, constitutes a highly promising class of electron acceptor materials with great potential for developing highly efficient and stable PSCs.

## Supplementary Material

nwaf537_Supplemental_File

## References

[1] Wang ZY, Bo YW, Bai PJ et al. Self-sustaining personal all-day thermoregulatory clothing using only sunlight. Science 2023; 382: 1291–6. 10.1126/science.adj3654 38096305

[2] Wang Z, Zhang D, Yang L et al. Mechanically robust and stretchable organic solar cells plasticized by small-molecule acceptors. Science 2025; 387: 381–7. 10.1126/science.adp9709 39847644

[3] Zhang GC, Lin FR, Qi F et al. Renewed prospects for organic photovoltaics. Chem Rev 2022; 122: 14180–274. 10.1021/acs.chemrev.1c00955 35929847

[4] Cheng P, Li G, Zhan XW et al. Next-generation organic photovoltaics based on non-fullerene acceptors. Nat Photon 2018; 12: 131–42. 10.1038/s41566-018-0104-9

[5] Meng LX, Zhang YM, Wan XJ et al. Organic and solution-processed tandem solar cells with 17.3% efficiency. Science 2018; 361: 1094–8. 10.1126/science.aat2612 30093603

[6] Lu X, Xie C, Liu Y et al. Increase in the efficiency and stability of large-area flexible organic photovoltaic modules via improved electrical contact. Nat Energy 2024; 9: 793–802. 10.1038/s41560-024-01501-1

[7] Wang JQ, Zheng Z, Bi PQ et al. Tandem organic solar cells with 20.6% efficiency enabled by reduced voltage losses. Natl Sci Rev 2023; 10: nwad085. 10.1093/nsr/nwad085 37448581 PMC10337743

[8] Son SY, Lee G, Wang HY et al. Integrating charge mobility, stability and stretchability within conjugated polymer films for stretchable multifunctional sensors. Nat Commun 2022; 13: 2739. 10.1038/s41467-022-30361-0 35585062 PMC9117230

[9] Yu H, Wang Y, Zou X et al. Effects of halogenation of small-molecule and polymeric acceptors for efficient organic solar cells. Adv Funct Mater 2023; 33: 2300712. 10.1002/adfm.202300712

[10] Zhang JQ, HS T, Guo XG et al. Material insights and challenges for non-fullerene organic solar cells based on small molecular acceptors. Nat Energy 2018; 3: 720–31. 10.1038/s41560-018-0181-5

[11] Yan CQ, Barlow S, Wang ZH et al. Non-fullerene acceptors for organic solar cells. Nat Rev Mater 2018; 3: 18003. 10.1038/natrevmats.2018.3

[12] Zuo LJ, Jo SB, Li YK et al. Dilution effect for highly efficient multiple-component organic solar cells. Nat Nanotechnol 2022; 17: 53–60. 10.1038/s41565-021-01011-1 34873302

[13] Zeng R, Zhang M, Wang XD et al. Achieving 19% efficiency in non-fused ring electron acceptor solar cells via solubility control of donor and acceptor crystallization. Nat Energy 2024; 9: 1117–28.

[14] Wen L, Mao HD, Zhang LF et al. Achieving desired pseudo-planar heterojunction organic solar cells via binary-dilution strategy. Adv Mater 2024; 36: 2308159. 10.1002/adma.202308159 37831921

[15] Lin YZ, Wang JY, Zhang ZG et al. An electron acceptor challenging fullerenes for efficient polymer solar cells. Adv Mater 2015; 27: 1170–4. 10.1002/adma.201404317 25580826

[16] Chen JD, Li YQ, Zhu JS et al. Polymer solar cells with 90% external quantum efficiency featuring an ideal light- and charge-manipulation layer. Adv Mater 2018; 30: 1706083. 10.1002/adma.201706083 29423980

[17] Cui Y, Zhang SQ, Liang NN et al. Toward efficient polymer solar cells processed by a solution-processed layer-by-layer approach. Adv Mater 2018; 30: 1802499. 10.1002/adma.201802499 29984486

[18] Zhang H, Yao H, Hou J et al. Over 14% efficiency in organic solar cells enabled by chlorinated nonfullerene small-molecule acceptors. Adv Mater 2018; 30: 1800613. 10.1002/adma.201800613 29806223

[19] Nian L, Kan YY, Wang HT et al. Ternary non-fullerene polymer solar cells with 13.51% efficiency and a record-high fill factor of 78.13%. Energy Environ Sci 2018; 11: 3392–9. 10.1039/C8EE01564C

[20] Yuan J, Zhang YQ, Zhou LY et al. Single-junction organic solar cell with over 15% efficiency using fused-ring acceptor with electron-deficient core. Joule 2019; 3: 1140–51. 10.1016/j.joule.2019.01.004

[21] Liu S, Yuan J, Deng WY et al. High-efficiency organic solar cells with low non-radiative recombination loss and low energetic disorder. Nat Photon 2020; 14: 300–5. 10.1038/s41566-019-0573-5

[22] Sun R, Wang T, Yang XR et al. High-speed sequential deposition of photoactive layers for organic solar cell manufacturing. Nat Energy 2022; 7: 1087–99. 10.1038/s41560-022-01140-4

[23] Zhang R, Chen HY, Wang TH et al. Equally high efficiencies of organic solar cells processed from different solvents reveal key factors for morphology control. Nat Energy 2025; 10: 124–34. 10.1038/s41560-024-01678-5

[24] Li C, Zhou JD, Song JL et al. Non-fullerene acceptors with branched side chains and improved molecular packing to exceed 18% efficiency in organic solar cells. Nat Energy 2021; 6: 605–13. 10.1038/s41560-021-00820-x

[25] Wang L, Chen C, Fu YW et al. Donor-acceptor mutually diluted heterojunctions for layer-by-layer fabrication of high-performance organic solar cells. Nat Energy 2024; 9: 208–18. 10.1038/s41560-023-01436-z

[26] Zhu L, Zhang M, Xu JQ et al. Single-junction organic solar cells with over 19% efficiency enabled by a refined double-fibril network morphology. Nat Mater 2022; 21: 656–63. 10.1038/s41563-022-01244-y 35513501

[27] Shi WD, Han QS, Zhu Y et al. A butterfly-shaped acceptor with rigid skeleton and unique assembly enables both efficient organic photovoltaics and high-speed organic photodetectors. Natl Sci Rev 2025; 12: nwae409. 10.1093/nsr/nwae409 39764497 PMC11702656

[28] Rong H, Ding P, Qin S et al. Reducing the trade-off between charge generation and nonradiative voltage loss via asymmetric strategy enables binary organic solar cells over 19.5%. ACS Energy Lett 2025; 10: 393–402. 10.1021/acsenergylett.4c03168

[29] Deng M, Xu X, Duan Y et al. 19.32% Efficiency polymer solar cells enabled by fine-tuning stacking modes of Y-type molecule acceptors: synergistic bromine and fluorine substitution of the end groups. Adv Mater 2024; 36: 2308216. 10.1002/adma.202308216 38100817

[30] Cho Y, Sun Z, Li GP et al. CF_3_-functionalized side chains in nonfullerene acceptors promote electrostatic interactions for highly efficient organic solar cells. J Am Chem Soc 2025; 147: 758–69. 10.1021/jacs.4c13471 39692398

[31] Dai T, Meng Y, Wang Z et al. Modulation of molecular quadrupole moments by phenyl side-chain fluorination for high-voltage and high-performance organic solar cells. J Am Chem Soc 2025; 147: 4631–42. 10.1021/jacs.4c17140 39841005

[32] Li C, Song JL, Lai HJ et al. Non-fullerene acceptors with high crystallinity and photoluminescence quantum yield enable >20% efficiency organic solar cells. Nat Mater 2025; 24: 433–43. 39880932 10.1038/s41563-024-02087-5

[33] Liu W, Wu W, Sergeev AA et al. Coplanar dimeric acceptors with bathochromic absorption and torsion-free backbones through precise fluorination enabling efficient organic photovoltaics with 18.63% efficiency. Adv Sci 2025; 12: 2410826. 10.1002/advs.202410826 PMC11904988 39834118

[34] Li Y, Ge ZW, Mei L et al. Isomeric dimer acceptors for stable organic solar cells with over 19% efficiency. Angew Chem Int Ed 2024; 63: e202411044. 10.1002/anie.202411044 39235423

[35] Ng HM, Zou B, Sergeev A et al. Improved efficiency and stability of outdoor and indoor organic photovoltaics with suppressed voltage loss via alkoxylation on dimeric giant acceptors featured as supramolecular stabilizers. Energy Environ Sci 2025; 18: 6587–96. 10.1039/D5EE00668F

[36] Liu W, Yuan J, Zhu C et al. A-π-A structured non-fullerene acceptors for stable organic solar cells with efficiency over 17%. Sci China Chem 2022; 65: 1374–82. 10.1007/s11426-022-1281-0

[37] Wang CX, Ma XM, Deng D et al. Giant dimeric donors for all-giant-oligomer organic solar cells with efficiency over 16% and superior photostability. Nat Commun 2024; 15: 8494. 10.1038/s41467-024-52821-5 39353930 PMC11445269

[38] Sun C, Lee JW, Lee CY et al. Dimerized small-molecule acceptors enable efficient and stable organic solar cells. Joule 2023; 7: 416–30. 10.1016/j.joule.2023.01.009

[39] Liu XC, Zhang Z, Wang C et al. A pyrene-fused dimerized acceptor for ternary organic solar cells with 19% efficiency and high thermal stability. Angew Chem Int Ed 2024; 63: e202316039. 10.1002/anie.202316039 37983686

[40] Yi F, Xiao MJ, Meng YD et al. Non-fully conjugated dimerized giant acceptors with different alkyl-linked sites for stable and 19.13% efficiency organic solar cells. Angew Chem Int Ed 2024; 63: e202319295. 10.1002/anie.202319295 38335036

[41] Song W, Ye QR, Yang SC et al. Ultra robust and highly efficient flexible organic solar cells with over 18% efficiency realized by incorporating a linker dimerized acceptor. Angew Chem Int Ed 2023; 62: e202310034. 10.1002/anie.202310034 37612732

[42] Wan J, Wang T, Sun R et al. Enabling highly efficient and thermal-stable polymer solar cells through semi-alloy acceptors composed of a hinge-like dimer: a versatile doping protocol. Adv Mater 2023; 35: 2302592. 10.1002/adma.202302592 37211895

[43] Shoaee S, Luong HM, Song J et al. What we have learnt from PM6:Y6. Adv Mater 2024; 36: 2302005. 10.1002/adma.202302005 37623325

[44] Perdigón-Toro L, Phuong LQ, Eller F et al. Understanding the role of order in Y-series non-fullerene solar cells to realize high open-circuit voltages. Adv Energy Mater 2022; 12: 2103422. 10.1002/aenm.202103422

[45] Guo Y, Han G, Yi Y. The intrinsic role of the fusion mode and electron-deficient core in fused-ring electron acceptors for organic photovoltaics. Angew Chem Int Ed 2022; 61: e202205975. 10.1002/anie.202205975 35604363

[46] Ma YL, Cai DD, Wan S et al. Ladder-type heteroheptacenes with different heterocycles for nonfullerene acceptors. Angew Chem Int Ed 2020; 59: 21627–33. 10.1002/anie.202007907 32790114

[47] Ma YL, Cai DD, Wan S et al. Control over π-π stacking of heteroheptacene-based nonfullerene acceptors for 16% efficiency polymer solar cells. Natl Sci Rev 2020; 7: 1886–95. 10.1093/nsr/nwaa189 34691530 PMC8288506

[48] Ma YL, Zhang M, Wan S et al. Efficient organic solar cells from molecular orientation control of M-Series acceptors. Joule 2021; 5: 197–209. 10.1016/j.joule.2020.11.006

[49] Xing K, Cai D, Wang D et al. Photovoltage enhancement of M-series acceptor-based polymer solar cells and minimodules through the modulation of charge-transfer states. Natl Sci Rev 2025; 12: nwaf089. 10.1093/nsr/nwaf089 40191256 PMC11970251

[50] Tu QS, Zheng WJ, Ma YL et al. Enhancing the intermolecular interactions of ladder-type heteroheptacene-based nonfullerene acceptors for efficient polymer solar cells by incorporating asymmetric side chains. CCS Chem 2023; 5: 455–68. 10.31635/ccschem.022.202101524

[51] Cai DD, Ma YL, Xing KC et al. Enhancing backbone organization and photovoltaic performance of M-series acceptors by using partially fluorinated side chains. Chem 2024; 10: 3131–47. 10.1016/j.chempr.2024.06.005

[52] Zhu YH, Ma YL, Liu L et al. Dimerized M-series acceptors with low diffusion coefficients for efficient and stable polymer solar cells. Angew Chem Int Ed 2024; 63: e202411155. 10.1002/anie.202411155 39160143

[53] Ghasemi M, Balar N, Peng ZX et al. A molecular interaction-diffusion framework for predicting organic solar cell stability. Nat Mater 2021; 20: 525–32. 10.1038/s41563-020-00872-6 33432145

[54] Gillett AJ, Privitera A, Dilmurat R et al. The role of charge recombination to triplet excitons in organic solar cells. Nature 2021; 597: 666–71. 10.1038/s41586-021-03840-5 34588666

[55] Zhu LY, Zhang JQ, Guo Y et al. Small exciton binding energies enabling direct charge photogeneration towards low-driving-force organic solar cells. Angew Chem Int Ed 2021; 60: 15348–53. 10.1002/anie.202105156 33942945

[56] Firdaus Y, Le Corre VM, Karuthedath S et al. Long-range exciton diffusion in molecular non-fullerene acceptors. Nat Commun 2020; 11: 5220. 10.1038/s41467-020-19029-9 33060574 PMC7562871

[57] Li Q, Wang R, Yu T et al. Long-range charge separation enabled by intramoiety delocalized excitations in copolymer donors in organic photovoltaic blends. J Phys Chem Lett 2023; 14: 7498–506. 10.1021/acs.jpclett.3c01861 37581453

[58] Wei X, Jia L, Duan B et al. Recent progress and applications of nanoIR-AFM in morphological characterization of organic solar cells. Adv Funct Mater 2024; 34: 2408960. 10.1002/adfm.202408960

