# Supplementary material for: Prolonged exciton lifetime via conjugation-length engineering in M-series acceptors for 19.39% efficiency polymer solar cells
Source: Natl Sci Rev. 2025 Nov 29;13(2):nwaf537. doi: 10.1093/nsr/nwaf537 (PMC12831031; doi:10.1093/nsr/nwaf537)
Supplement: nwaf537_Supplemental_File [file nwaf537_supplemental_file.pdf]

Supplementary information

Prolonged exciton lifetime *via* conjugation-length engineering in M-Series acceptors for 19.39% efficiency polymer solar cells

Wenxiong Shen,[1,†] Xiaoying Xiong,[1,†] Dongdong Cai,[2,†] Li Liu,[2] Junlu Lin,[3] Shuo Wan,[1] Jin-Yun Wang,[2] Yi Li,[1] Yunlong Ma,[2,*] Huiting Fu,[1] Chunfeng Zhang[3] and Qingdong Zheng[1,*]

[1]State Key Laboratory of Coordination Chemistry, College of Engineering and Applied Sciences, Nanjing University, Nanjing 210023, China

[2]State Key Laboratory of Structural Chemistry, Fujian Institute of Research on the Structure of Matter, Chinese Academy of Sciences, Fuzhou 350002, China

[3]National Laboratory of Solid State Microstructures, School of Physics, and Collaborative Innovation Center for Advanced Microstructures, Nanjing University, Nanjing 210093, China

***Corresponding authors**. E-mails: zhengqd@nju.edu.cn; mayunlong@fjirsm.ac.cn

[†]Equally contributed to this work.

Supplementary Notes 1-17

Supplementary Figures S1-22

Supplementary Tables S1-14

Supplementary Notes

Supplementary Note 1. Materials and instruments

The synthesis of 2-(5-bromo-4,6-difluoro-3-oxo-2,3-dihydro-1H-inden-1-ylidene)malononitrile (IC-2FBr), 2-(5-bromo-4,6-dichloro-3-oxo-2,3-dihydro-1H-inden-1-ylidene) malononitrile (IC-2ClBr), compound **1** and the piperazine-functionalized derivative of perylene diimide (PDIP) were synthesized according to procedures previously reported in our work [1-5]. PM6, 2-(5,6-difluoro-3-oxo-2,3-dihydro-1H-inden-1-ylidene)malononitrile, 2-(5,6-dichloro-3-oxo-2,3-dihydro-1H-inden-1-ylidene)malononitrile, (2-(9H-carbazol-9-yl)ethyl)phosphonic acid (2PACz), poly[(9,9-bis(3′-(N,N-dimethylamino)propyl)-2,7-fluorene)-*alt*-5,5′-bis(2,2′-thiophene)-2,6-naphthalene-1,4,5,8-tetracaboxylic-N,N′-di(2-ethylhexyl)imide] (PNDIT-F3N), and N,N'-bis[3-(dimethylamino)propyl] perylene-3,4,9,10-tetracarboxylic diimide (PDIN) were purchased from Solarmer Materials Inc. and Suna Tech Inc., respectively. 1-Chloronaphthalene (CN), 2-chloronaphthalene (2-CN), 4-bromochlorobenzene (BCB) and trichlorobenzene (TCB) were purchased from TCI. 2-Methoxynaphthalene (2-MN), 2-ethoxynaphthalene (2-EN), 2-propoxynaphthalene (2-PN) were purchased from Aladdin. 1,3-Dibromo-5-chlorobenzene (DBCB), and 3,5-dichlorobromobenzene (DCBB) were purchased from Macklin Inc. 1,3,5-Tribromobenzene (TBB) and 1,4-dichlorobenzene (DCB) were purchased from Bide Pharma tech Ltd. All other solvents and reagents were obtained from Adamas-beta Ltd., Suna Tech Inc., and Energy Chemical, and were used directly without further purification unless otherwise stated. Nuclear magnetic resonance (NMR) spectra were recorded on a Bruker AVANCE-400 spectrometer using deuterated chloroform as the solvent and tetramethylsilane (TMS) as the internal standard. High-resolution mass spectrometry (HRMS) measurements were obtained using a UHR TOF LC/MS Mass Spectrometer. Absorption spectra were acquired using a Lambda 365 UV-vis spectrophotometer. The surface morphology of the blend films was characterized by atomic force microscopy (AFM) in Peak Force Quantitative Nanomechanical Mapping mode.

Supplementary Note 2. Synthesis and characterization

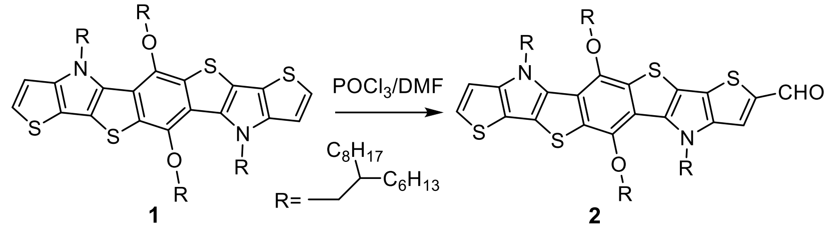

Synthesis of Compound 2: In a dry two-neck round-bottom flask, Compound **1** (2.62 g, 2.00 mmol) was dissolved in 35 mL of 1,2-dichloroethane and placed under a nitrogen atmosphere. The solution was cooled in an ice bath while phosphorus oxychloride (2.45 g, 16 mmol) and DMF (1.17 g, 16 mmol) were added successively. The reaction mixture was stirred for 1 hour at room temperature. After completion of the reaction, the mixture was poured into ice water, neutralized with Na_2CO_3, and then extracted with dichloromethane. The combined organic layers were washed with water and brine, then dried over anhydrous $MgSO_4$. After evaporation of the solvent under reduced pressure, the crude product was purified by column chromatography on silica gel using a petroleum ether/dichloromethane (2:1) eluent to yield compound **2** as a dark blue oil (2.22 g, 83%). ^{1}H NMR (400 MHz, $CDCl_3$, ppm) : δ 9.93 (s, 1H), 7.70 (s, 1H), 7.24 (d, J = 5.2 Hz, 1H), 7.08 (d, J = 5.2 Hz, 1H), 4.75 (d, J =7.5 Hz, 2H), 4.70 (d, J =7.5 Hz, 2H), 4.05 (d, J =6.9 Hz, 2H), 3.98 (d, J =6.9 Hz, 2H), 2.15-1.96 (m, 4H), 1.66 (m, 4H), 1.52-0.74 (m, 116H). HRMS (MALDI) m/z: calcd. for $C_{83}H_{136}N_2O_3S_4$, 1336.9428; found, 1336.9386.

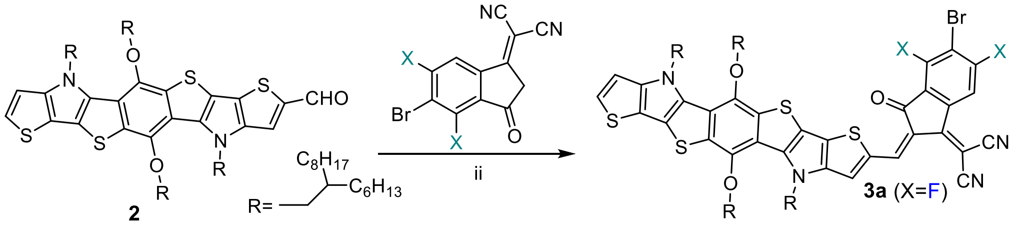

Synthesis of Compound 3a: Compound **2** (1.00 g, 0.75 mmol) and IC-2FBr (0.92 g, 2.99 mmol) were dissolved in anhydrous chloroform (20 mL) in a two-necked round-bottom flask. The mixture was deoxygenated with nitrogen for 30 minutes, followed by the addition of 0.2 mL of pyridine. The reaction mixture was stirred at reflux for 6 hours under a nitrogen atmosphere. After completion of the reaction, the mixture was poured into methanol, and the resulting precipitate was filtered off. The crude product was purified by column chromatography on silica gel using petroleum ether/dichloromethane (3:2) as the eluent, yielding compound **3a** as a dark blue oil (0.89 g, 73%).

^{1}H NMR (400 MHz, $CDCl_3$, ppm): δ 8.93 (s, 1H), 8.25 (d, J = 8.1 Hz, 1H), 7.74 -7.26 (m, 2H), 7.09 (d, J = 5.2 Hz, 1H), 4.75 (d, J =7.5 Hz, 2H), 4.70 (d, J =7.5 Hz, 2H), 4.00 (d, J =7.1 Hz, 2H), 3.97 (d, J =7.1 Hz, 2H), 2.16-1.96 (m, 4H), 1.66 (m, 4H), 1.52-0.74 (m, 116H). HRMS (MALDI) m/z: calcd. for $C_{95}H_{137}N_4O_3S_4F_2Br$, 1626.8719; found, 1626.8678.

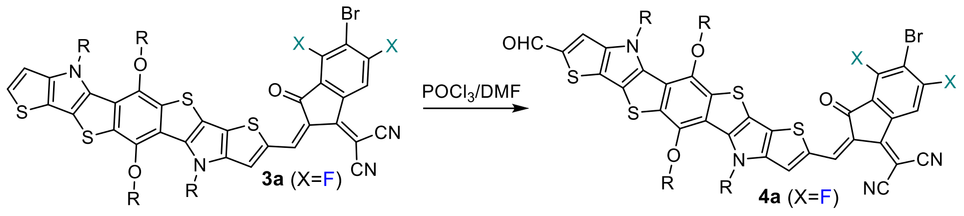

Synthesis of Compound 4a: In a dry two-neck round-bottom flask, Compound **3a** (1.63 g, 1.00 mmol) was dissolved in 35 mL of 1,2-dichloroethane and placed under a nitrogen atmosphere. The solution was cooled in an ice bath and stirred, while phosphorus oxychloride (1.23 g, 8 mmol) and DMF (0.59 g, 8 mmol) were added successively. The mixture was then stirred at reflux for 1 hour under nitrogen. After the reaction, the mixture was poured into ice water and neutralized with Na_2CO_3, followed by extraction with dichloromethane. The combined organic layer was washed with water and brine, and dried over anhydrous $MgSO_4$. After evaporating the solvent under reduced pressure, the crude product was purified by column chromatography on silica gel (petroleum ether: dichloromethane = 2:1) to yield compound **4a** as a dark blue oil (1.48 g, 89%). ^{1}H NMR (400 MHz, $CDCl_3$, ppm): δ 9.96 (s, 1H), 8.98 (s, 1H), 8.32 (d, J = 7.8 Hz, 1H), 7.89 (br, 1H), 7.73 (s, 1H), 4.74 (m, 4H), 4.03-3.98 (m, 4H), 2.12-1.99 (m, 4H), 1.64 (m, 4H), 1.52-0.74 (m, 116H). Calcd. for $C_{96}H_{137}N_4O_4S_4F_2Br$, 1654.8669; found, 1654.8623.

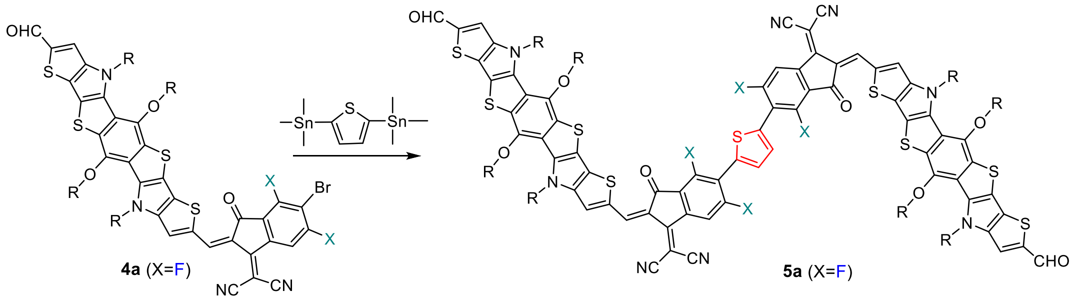

Synthesis of Compound 5a: A mixture of Compound **4a** (3.31 g, 2.00 mmol), 2,5-bis(trimethylstannyl)thiophene (0.37 g, 0.90 mmol), $Pd_2(dba)_3$ (92 mg, 0.10 mmol), and P(o-tolyl)$_3$ (122 mg, 0.40 mmol) was placed in a sealed, thick-walled pressure flask under a nitrogen atmosphere. Then, 10 mL of toluene, bubbled with nitrogen, was added to the reaction mixture. The

reaction was allowed to proceed at reflux. After completion, the mixture was poured into ice water and extracted with dichloromethane. The combined organic layers were washed with water and brine, and then dried over $MgSO_4$. After the solvent was removed under reduced pressure, the crude product was purified by silica gel column chromatography (eluent: petroleum ether: dichloromethane = 2:1) to yield Compound **5a** as a dark blue solid (2.10 g, 65%). ^1H NMR (400 MHz, $CDCl_3$, ppm): δ 9.95 (s, 2H), 8.96 (s, 2H), 8.38 (d, J = 10.7 Hz, 2H), 7.90 (s, 4H), 7.71 (br, 2H), 4.74 (d, J = 7.6 Hz, 8H), 4.00 (br, 8H), 2.12-1.99 (m, 8H), 1.64 (br, 8H), 1.52-0.73 (m, 232H). HRMS (MALDI) m/z: calcd. for $C_{196}H_{276}N_8O_8S_9F_4$, 3233.8853; found, 3233.8848.

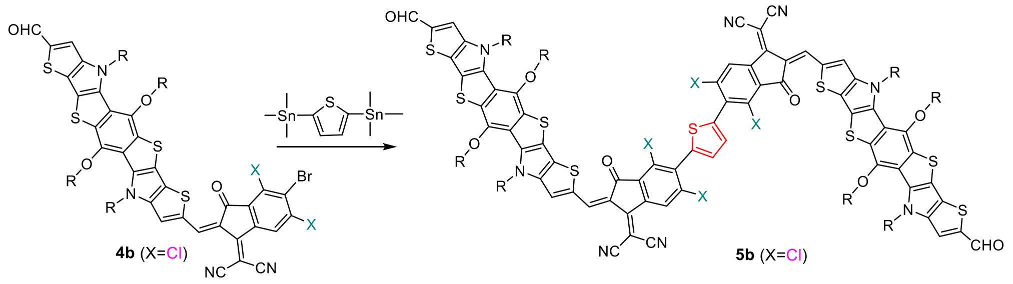

Synthesis of Compound 5b: Compound **5b** was synthesized using the same procedure as that for **5a**. It was isolated as a dark blue solid in 72% yield by silica gel chromatography with n-hexane:CH_2Cl_2 (1:1) as the eluent. ^1H NMR (400 MHz, $CDCl_3$): δ 9.96 (s, 2H), 9.06 (s, 2H), 8.81 (s, 2H), 7.85 (m, 2H), 7.73 (s, 2H), 7.18 (s, 2H), 4.74 (d, J = 6.8 Hz, 8H), 4.01 (m, 8H), 2.12 (m, 4H), 1.99 (m, 4H), 1.39-0.99 (m, 192H), 0.91-0.86 (m, 24H), 0.83-0.74 (m, 24H). HRMS (MALDI) m/z: calcd. for $C_{196}H_{276}Cl_4N_8O_8S_9$, 3297.7671; found, 3297.7778.

Synthesis of DM-8F: Compound **5a** (0.10 g, 0.031 mmol) and 2-(5,6-difluoro-3-oxo-2,3-dihydro-1H-inden-1-ylidene)malononitrile (15 mg, 0.065 mmol) were dissolved in 35 mL of toluene, followed by slow addition of acetic anhydride (0.06 mL) and boron trifluoride etherate (88 mg, 0.62 mmol) under stirring. The resulting mixture was stirred at room temperature for 1 hour under a nitrogen atmosphere. Upon completion of the reaction, the mixture was carefully poured into methanol, resulting in the formation of a precipitate, which was subsequently collected by filtration. The crude product was purified by column chromatography on silica gel (eluent: petroleum ether/dichloromethane = 2/1) to yield DM-8F as a dark blue solid (0.08 g, 88%). Solubility: 36 mg/mL in chloroform at room temperature. ^1H NMR (400 MHz, $CDCl_3$, ppm): δ 8.94 (s, 2H), 8.90 (s, 2H), 8.50-8.54 (m, 2H), 8.37 (d, J = 11.6 Hz, 2H), 7.90-7.83 (m, 6H), 7.69 (t, J = 7.8 Hz, 2H), 4.76 (br, 8H), 4.05 (br, 8H), 2.15 (br, 4H), 2.00 (br, 4H), 1.70 (br, 8H), 1.43-0.74 (m, 232H). HRMS

(MALDI) m/z: calcd. for $C_{220}H_{280}F_8N_{12}O_8S_9$, 3657.9225; found, 3657.9224.

Synthesis of DM-8Cl: DM-8Cl was synthesized by the reaction between Compound **5b** and 2-(5,6-dichloro-3-oxo-2,3-dihydro-1H-inden-1-ylidene)malononitrile using the same procedure as that for DM-8F. The crude product was then purified by silica gel column using petroleum ether/dichloromethane (1:1 by volume) as the eluent, yielding a dark blue solid (85% yield). Solubility: 20 mg/mL in chloroform at room temperature. ^{1}H NMR (400 MHz, $CDCl_3$): δ 9.06 (s, 2H), 8.98 (s, 2H), 8.82 (s, 2H), 8.78 (s, 2H), 7.96 (m, 4H), 7.85 (m, 2H), 7.19 (m, 2H), 4.76 (d, *J* = 6.8 Hz, 8H), 4.02 (m, 8H), 2.12 (m, 4H), 2.00 (m, 4H), 1.39-0.99 (m, 192H), 0.91-0.86 (m, 24H), 0.83-0.74 (m, 24H). HRMS (MALDI) m/z: calcd. for $C_{220}H_{280}Cl_8N_{12}Os_8S_9$, 3788.6910; found, 3788.6996.

Synthesis of M68: The synthesis of M68 followed a protocol analogous to that employed for the typical M-series small molecule acceptors reported in our previous studies [2]. Solubility: 59 mg/mL in chloroform at room temperature. ^{1}H NMR (400 MHz, $CDCl_3$): δ 8.95 (s, 2H), 8.53 (m, 2H), 7.91 (s, 2H), 7.71 (t, *J* = 7.5 Hz, 2H), 4.72 (d, *J* = 7.8 Hz, 4H), 4.01 (d, *J* = 6.9 Hz, 4H), 2.13-1.98 (m, 4H), 1.72-0.71 (m, 120H). HRMS (MALDI) m/z: calcd. for $C_{108}H_{140}N_6O_4F_4S_4$, 1788.9750; found, 1788.9772.

Supplementary Note 3. Fabrication of small-area devices and minimodule devices

The conventional devices were constructed with the following structure: ITO/2PACz/active layer/PNDIT-F3N/Ag. The pre-patterned ITO glass substrates, which have a sheet resistance of 15 Ω, were sequentially sonicated in detergent, deionized water, ethanol, and isopropyl alcohol for 30 minutes each. They were then dried at 70 °C before use. Subsequently, all substrates were exposed to ultraviolet/ozone for 15 minutes. 2PACz solution (0.5 mg/mL in methanol) was spin-coated on the ITO substrates at 2000 rpm for 30 seconds, followed by annealing at 90 °C for 10 minutes. For the preparation of traditional bulk heterojunction (BHJ) devices, photovoltaic materials are dissolved in chloroform with a specific amount of 1-chloronaphthalene to achieve a concentration of 15 mg/mL, maintaining a donor/acceptor weight ratio of 1:1. The blend solution was spin-coated onto the substrate at 2500 rpm to achieve an approximately 100 nm thick film in a nitrogen-filled glove box. For the active layer processed using layer-by-layer (LBL) deposition, the concentration

of PM6 is 9.5 mg/mL in chlorobenzene. And the concentrations of the dimerized acceptor (DM-8F or DM-8Cl) and small molecule acceptor M68 are 6.5 mg/mL and 7.5 mg/mL in chloroform, respectively. For the optimal device performance, the donor solution was first spin-coated at 1800 rpm, succeeded by the acceptor solution, which underwent spin-coating at 2000 rpm for a duration of 30 seconds. Finally, a 150 nm thick silver (Ag) layer was deposited onto the PNDIT-F3N electron-transporting layer *via* thermal evaporation under a pressure of 1.0×10^{-4} Pa. The devices were fabricated with an active area of 4.2 mm^2.

Module devices were fabricated with a structure of ITO/PEDOT:PSS/BHJ active layer/PDIN/Ag following the procedure outlined below. The patterned ITO substrates (10 Ω, with a maximum transmittance of approximately 89%) were cleaned as previously described. The pre-cleaned substrates were treated in an ultraviolet-ozone chamber for 15 minutes. A thin film of poly(3,4-ethylenedioxythiophene):poly(styrenesulfonate) (PEDOT:PSS, Clevios PVP AI 4083) was deposited onto the ITO substrate *via* spin-coating, yielding a thickness of approximately 20 nm, followed by thermal annealing at 150 °C for 15 minutes in ambient atmosphere. Later, a blend solution of PM6:DM-8F (1:1, w/w, 14.0 mg/mL in chlorobenzene) was spin-coated at 2300 rpm for 40 seconds to form the active layer, which was subsequently annealed at 100 °C for 20 minutes. A PDIN layer, approximately 10 nm in thickness, was spin-coated onto the active layer from a 2.0 mg/mL methanol solution. A mechanical scribe with a pointed cotton swab was used to create the P2 patterns. The module devices were then placed in an evaporation chamber, where a 150 nm thick silver layer was thermally evaporated onto the PDIN layer at a pressure of 1.0×10^{-4} Pa. Each module device consists of four sub-cells, with each sub-cell having an active area of 2.773 cm^2. The total active layer area for the efficiency test is 11.09 cm^2.

Supplementary Note 4. Characterization of and stability test of PSCs

Current density-voltage (*J-V*) characteristics were recorded in a glove box using a Keithley 2400 Source Measure unit at room temperature. The photocurrent was measured under AM 1.5G illumination at 100 mW/cm^2 with a solar simulator (Enlitech, SS-F5). The light intensity was calibrated using a standard silicon solar cell. The external quantum efficiency (EQE) spectra were obtained from a QEX10 Solar Cell EQE measurement system (PV Measurement, Inc.) For the thermal stability evaluation, unencapsulated inverted devices with a structure of ITO/ZnO/BHJ

active layer/MoO_3/Ag were heated at 80 °C on a hotplate under the open-circuit conditions in a N_2-filled glove box. The ZnO layer was prepared according to the previous work [6], and the initial PCEs of PM6:M68-, PM6:DM-8F-, and PM6:DM-8Cl-based devices are 15.2%, 17.4%, and 14.5%, respectively. For the light stability evaluation, the devices were fabricated with a structure of ITO/MoO_3/2PACz/LBL active layer/PDIP/Ag. ITO/MoO_3 substrate was prepared by evaporating 5 nm of MoO_3 on the ultraviolet/ozone-treated ITO, and the initial PCEs of PM6:M68-, PM6:DM-8F-, and PM6:DM-8Cl-based devices are 17.2%, 18.8%, and 16.5%, respectively. The stability test was tracked under continuous illumination at maximum power point (MPP) using a light-emitting diode array at 25 °C, which had a light intensity of approximately 1 Sun.

Supplementary Note 5. Cyclic voltammetry measurements

Cyclic voltammetry (CV) measurements were conducted using a CHI 604E electrochemical workstation with a three-electrode cell, in a nitrogen-bubbled 0.1 M tetrabutylammonium hexafluorophosphate (Bu_4NPF_6) solution in acetonitrile, at a scan rate of 100 mV s^{-1} and room temperature. Platinum wire, Ag/$AgNO_3$ (0.1 M $AgNO_3$ in acetonitrile), and a platinum plate were used as the counter electrode, reference electrode, and working electrode, respectively. The Ag/$AgNO_3$ reference electrode was calibrated with a ferrocene/ferrocenium redox couple as the external standard, with the oxidation potential set at -4.82 eV relative to the zero-vacuum level. The dimerized acceptor films were deposited onto a platinum (Pt) plate electrode by dipping the electrode into the corresponding solutions and allowing them to dry. The HOMO/LUMO energy levels of the materials were calculated according to the following equations:

$$E_{\mathrm{HOMO/LUMO}} = -(\varphi_{\mathrm{ox}}/\varphi_{\mathrm{red}} + 4.82)\ (\mathrm{eV})$$

where φ_{ox} and φ_{red} are the onset oxidation and reduction potentials relative to the Ag/$AgNO_3$ reference electrode, respectively.

Supplementary Note 6. Estimation of glass-transition temperature

The T_g was measured by monitoring changes in the UV-Vis absorption characteristics of organic films after annealing. The ordered aggregation of organic films is the main cause of these changes, and the UV-Vis absorption spectrum undergoes significant alterations once the annealing temperature exceeds the T_g of the material. We used the same process conditions (i.e., solvent,

concentration, and spin-coating speed) as for the best-performing devices to fabricate films of M68 and two dimerized acceptors. The UV-Vis absorption spectra of the acceptor films were measured at increasing temperatures from room temperature (RT) to 240 °C. The annealing time for each film was 5 minutes, and the films were allowed to cool to RT before the UV-Vis absorption measurements. For quantitative analysis, the deviation metric (DMT) was calculated for each absorption spectrum according to the method reported by Samuel E. Root *et al.* [7], $\mathrm{DM}_T = \sum_{\lambda_{\min}}^{\lambda_{\max}}[I_{\mathrm{RT}}(\lambda) - \mathrm{I_T}(\lambda)]^2$, where λ is the wavelength, λ_{max} and λ_{min} are the upper and lower bounds of the optical sweep, respectively, and $I_{RT}(\lambda)$ and $I_T(\lambda)$ represent the normalized absorption intensities of the film at RT and after annealing, respectively. The intersection of the two fitted lines in the low- and high-temperature regions indicates T_g.

Supplementary Note 7. Estimation of diffusion coefficient at 85 °C (D_{85})

The D_{85} values of the acceptor constituents in blend films with PM6 were estimated using the method outlined in previous literature (i.e., the Ghasemi-O'Connor-Ade framework) [8]. It was observed that there is a significant correlation between the D_{85} of acceptor materials blended with specific donor materials and their glass transition temperatures (T_gs). Specifically, as the T_g of the material increases, D_{85} decreases exponentially, following the relationship: D_{85} (cm^2 s^{-1}) = a×e$^{b\times T_g(K)}$, where the coefficients a and b are specific to the donor material. For blends with PM6 as the donor, the D_{85} of the acceptor can be calculated using the following equation: D_{85} (cm^2 s^{-1}) = 1.2×10^7×e$^{-0.15\times T_g(K)}$.

Supplementary Note 8. Highly sensitive EQE and EQE$_{EL}$ measurements

Highly sensitive EQE (s-EQE) was measured using an integrated system (PECT-600, Enlitech). External quantum efficiency of electroluminescence (EQE$_{EL}$) and electroluminescence (EL) spectra were collected by applying external voltage (1-3 V) through the devices (ELCT-3010, Enlitech). All devices for EQE$_{EL}$ measurements were prepared according to the optimal device fabrication conditions.

Supplementary Note 9. Calculation of the energy loss

The detailed calculation of energy loss is presented as follows [9]:

$$V_{oc}^{SQ} = \frac{kT}{q}\ln\left(\frac{J_{SC}}{J_0^{SQ}}+1\right) = \frac{kT}{q}\ln\left(\frac{q\cdot\int_0^{\infty} EQE_{PV}(E)\cdot\Phi_{AM1.5}(E)dE}{q\cdot\int_{E_{gap}}^{\infty}\Phi_{BB}(E)dE}+1\right)$$

$$V_{oc}^{rad} = \frac{kT}{q}\ln\left(\frac{J_{SC}}{J_0^{rad}}+1\right) = \frac{kT}{q}\ln\left(\frac{q\cdot\int_0^{\infty} EQE_{PV}(E)\cdot\Phi_{AM1.5}(E)dE}{q\cdot\int_0^{\infty} EQE_{PV}(E)\cdot\Phi_{BB}(E)dE}+1\right)$$

$$\Phi_{BB}(E) = \frac{2\pi}{h^3c^2}E^2\exp(-\frac{E}{KT})$$

$$\Delta E_1 = E_g\text{-}V_{oc}^{SQ}$$

$$\Delta E_2 = V_{oc}^{SQ} - V_{oc}^{rad}$$

$$\Delta E_3 = -\frac{kT}{q}\ln EQE_{EL}$$

Where q is the elementary charge, V_{oc}^{SQ} is the maximum voltage in the Shockley-Queisser (SQ) limit model, and V_{oc}^{rad} is the V_{oc} with only radiative recombination in the device. The integral boundaries a and b are selected where $P(a) = P(b) = 0.5\max[P(E_g)]$. The selection of integral boundaries serves to exclude the influence of noisy data and negative value of $P(E_g)$, and is not physically motivated. While the factor 0.5 in the choice of a and b is fairly arbitrary, slightly different choices would not strongly affect the result except for very noisy data.

Supplementary Note 10. Determination of dielectric properties

The dielectric constants of the neat films and the blend films were determined by the capacitance-frequency measurements with a device structure of Al/the neat films or the blend films/Al under the frequency from 20 Hz to 2.0×10^5 Hz. The dielectric constant was evaluated by the material's geometric capacitance [10]. The geometric capacitance, C_g, was used to calculate the dielectric constant using the following equation [11]: $\varepsilon = \frac{C_g d}{\varepsilon_0 A}$, where the d is the active layer thickness, ε_0 is the vacuum permittivity (8.85×10^{-12} F m^{-1}), and A is the contact area. The active layer area of the device is 0.09 cm^2.

Supplementary Note 11. Temperature-dependent photoluminescence measurements

To demonstrate the contribution of improved ε_r to the E_a, we conducted a temperature-dependent photoluminescence (PL) spectra were measured to obtain the experimental E_a. Solutions of two

dimerized acceptors and M68 (15 mg/mL) were prepared in chloroform, and thin films were fabricated by spin-coating the prepared solutions onto quartz substrates. Temperature-dependent PL spectra were measured using an Edinburgh Instrument FLS 980 multi-function fluorescence spectrometer, with excitation at a wavelength of 750 nm for M68 and two dimerized acceptors, provided by an ozone-free continuous xenon lamp. Thermal quenching of PL emission intensity was observed from 80 to 300 K. The E_a was calculated by fitting the integrated PL emissions as a function of temperature according to the Arrhenius equation [12-14]: $I(T) = \frac{I_0}{1 + \mathrm{A}\exp\left(-E_a/k_B T\right)}$, where I_0 is the intensity at 0 K, k_B is the Boltzmann constant, and T is the temperature.

Supplementary Note 12. Charge exciton dissociation analysis

To study the exciton dissociation processes of the photovoltaic devices, plots of the J_{ph} *versus* V_{eff} of the PSCs were measured [15]. Here, J_{ph} and V_{eff} are defined as $J_{\mathrm{ph}} = J_{\mathrm{L}} - J_{\mathrm{D}}$ and $V_{\mathrm{eff}} = V_0 - V_{\mathrm{appl}}$, respectively. In these expressions, J_{D} and J_{L} are the photocurrent densities in the dark and under the illumination, respectively, while V_{appl} is the applied bias voltage, and V_0 is the voltage at which $J_{\mathrm{ph}} = 0$. Usually, V_{eff} determines the electric field in the bulk region and thereby determines the carrier transport and the photocurrent extraction. At high V_{eff} values, charge carriers rapidly move toward the related electrodes with minimal recombination. The J_{ph} reaches the saturation current density (J_{sat}) at a high V_{eff} ($\geq$ 2.0 V in these cases).

Supplementary Note 13. Hole and electron mobilities characterization

Hole and electron mobilities were measured using the space-charge-limited-current (SCLC) method. Hole-only devices were fabricated with ITO/2PACz/active layer/MoO_3/Ag, while electron-only devices were constructed with ITO/ZnO/active layer/PNDIT-F3N/Al architecture. The active layers were prepared using the same method for the best-performance OSC fabrication. Device areas were fixed at 4.2 mm^2. A Keithley 2400 source measurement unit measured the current density (J). The SCLC hole/electron mobilities were calculated according to the following equation:

$$J = \frac{9\varepsilon_r \varepsilon_0 \mu V^2}{8L^3}$$

Where J is the current density (A m^{-2}), ε_0 is the free-space permittivity (8.85×10^{-12} F m^{-1}), and ε_r is the relative dielectric constant of the active layer material, usually 2-4 for organic semiconductors,

herein we used a relative dielectric constant of 3, μ is the mobility of hole or electron, V is the voltage drop across the SCLC device ($V = V_{app}$-V_{bi}, where V_{app} is the applied voltage to the device and V_{bi} is the built-in voltage due to the difference in the work function of two electrodes, in the hole- and electron-only devices, the V_{bi} values are 0.5 and 0.7 V, respectively), and L is the thickness of the active layer. The film thickness was detected by a Bruker Dektak XT surface profilometer. The hole- and electron mobilities were calculated from the slopes of the $J^{1/2}$-V curve.

Supplementary Note 14. Transient absorption instruments

Ultrafast broadband transient absorption (TA) spectroscopy was performed using a Yb:KGW laser (Pharos, Light Conversion). A homemade noncollinear optical parametric amplifier (NOPA), pumped by the second harmonic generation of the fundamental frequency light, was employed to generate the pump beam. The pump wavelength was tuned to 860 nm to selectively excite the acceptor. The probe light was generated via white-light supercontinuum generated by focusing a small fraction of the 1030 nm fundamental beam onto a 5-mm-thick sapphire plate. To ensure isotropic response measurements, the polarization of the pump and probe beams was set at the magic angle (54.7°). The instrument response function (IRF) of the setup was approximately 100 fs. The excitation fluence at the sample was maintained at ~0.5 μJ/cm² unless otherwise stated.

Supplementary Note 15. Calculation processes of exciton diffusion length

When exciton-exciton annihilation (EEA) occurs in the film, the exciton decay dynamics accelerate with increasing excitation fluences [16]. In this study, we employed excitation fluences ranging from 0.5 to 16 μJ/cm². The exciton decay kinetics were globally fitted using a rate equation that accounts for both exciton annihilation and the first-order recombination:

$$\frac{\mathrm{d}n(t)}{\mathrm{d}t} = -\kappa n(t) - \frac{1}{2}\alpha n^2(t)$$

which has the analytical solution:

$$n(t) = \frac{n(0)e^{-kt}}{1 + \frac{\alpha}{2\kappa}n(0)(1 - e^{-kt})}$$

where κ is the intrinsic exciton decay rate constant, and α represents the bimolecular exciton-exciton annihilation rate. The value of κ is extracted by fitting the kinetic decay at the lowest excitation fluence, while α is determined by fitting the decay traces at varying excitation fluences.

Then, the exciton diffusion coefficient (D) and diffusion length (L_{D}) are calculated using the following expressions:

$$D = \frac{\alpha}{8\pi R}$$

$$L_D = (\frac{D}{\kappa})^{\frac{1}{2}}$$

where R is the singlet exciton annihilation radius, typically assumed to be 1 nm.

Supplementary Note 16. GIWAXS characterization

The 2D GIWAXS patterns were obtained using a XEUSS SAXS/WAXS system at the Fujian Science & Technology Innovation Laboratory for Optoelectronic Information, China. All samples for the GIWAXS measurements were prepared on PEDOT:PSS-coated Si substrates following the same fabrication method as that used for the best-performance OSCs. The X-ray beam had a wavelength of 1.54 Å, with the incident angle set at 0.2°. Scattered X-rays were detected using a Dectris Pilatus 300 K photon-counting detector.

Supplementary Note 17. Photo-induced force microscope (PiFM)

Photo-induced force microscopy (PiFM) results were acquired using a VistaScope microscope from Molecular Vista, Inc. All PiFM experiments were excited by a pulsed quantum cascade laser (Block Engineering) with a gap-free narrowband tunable wavenumber of 760-1950 cm^{-1}. The spectral linewidth is ~2 cm^{-1} with a wavenumber resolution of 0.5 cm^{-1}. The PiFM experiment here was operated at the sideband excitation with the laser-frequency modulated at $f_m=f_1-f_0$, where f_0 is the first mechanical eigenmode resonances of the cantilever that is used for PiFM signal detection, while f_1 denotes the second ones recorded for the AFM topography of the sample. The probe is a Pt-coated tip with a resonant frequency of ~350 kHz (PPP-NCHPt-MB, Nanosensors).

Supplementary Figures

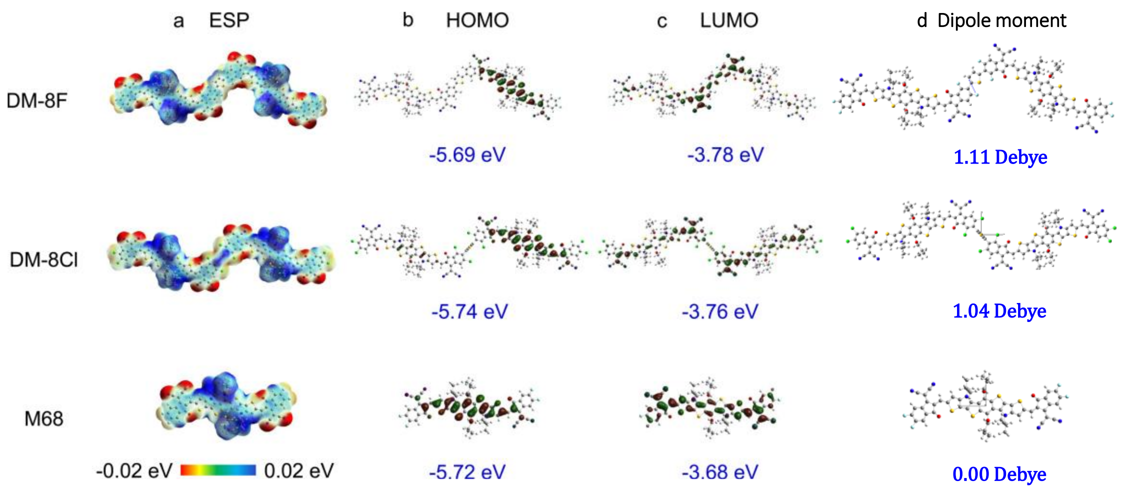

Supplementary Figure S1. (a) Surface ESP distributions of DM-8F, DM-8Cl, and M68; Calculated (b) HOMO, (c) LUMO energy levels and (d) dipole moments of DM-8F, DM-8Cl, and M68.

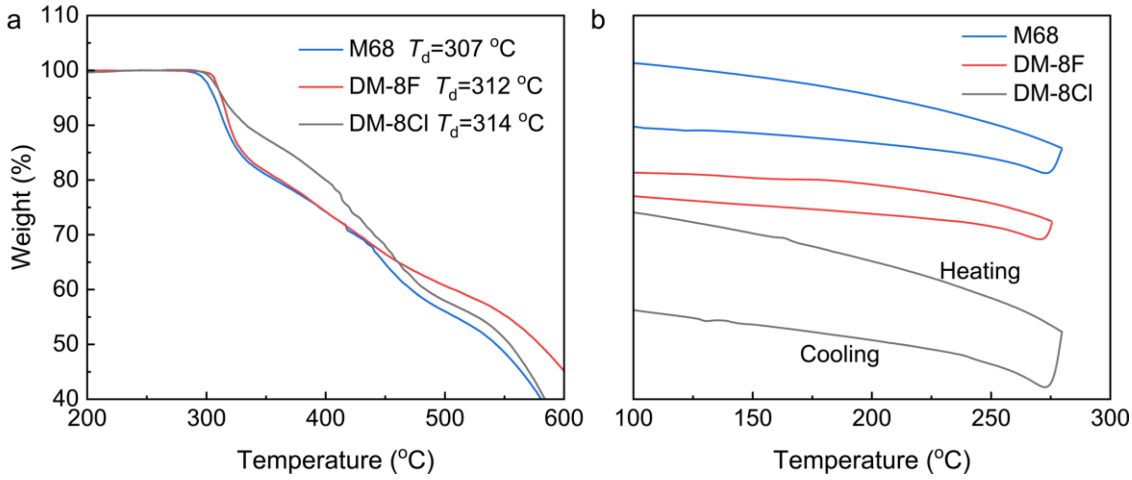

Supplementary Figure S2. (a) The TGA and (b) DSC (2nd heating cycles) curves of DM-8F, DM-8Cl, and M68.

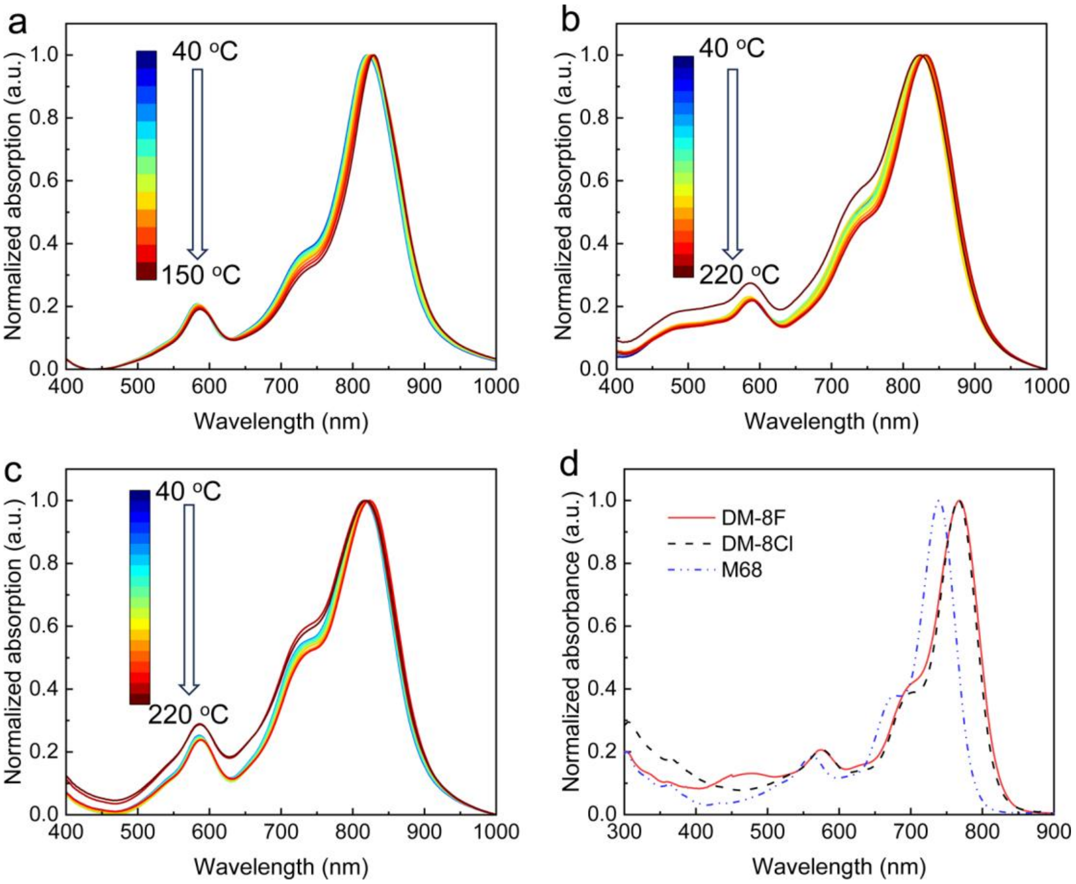

Supplementary Figure S3. Normalized absorption spectra of the (a) M68, (b) DM-8F, and (c) DM-8Cl films at different annealing temperatures. (d) Normalized UV-vis-NIR absorption spectra of M68, DM-8F, and DM-8Cl in chloroform solution.

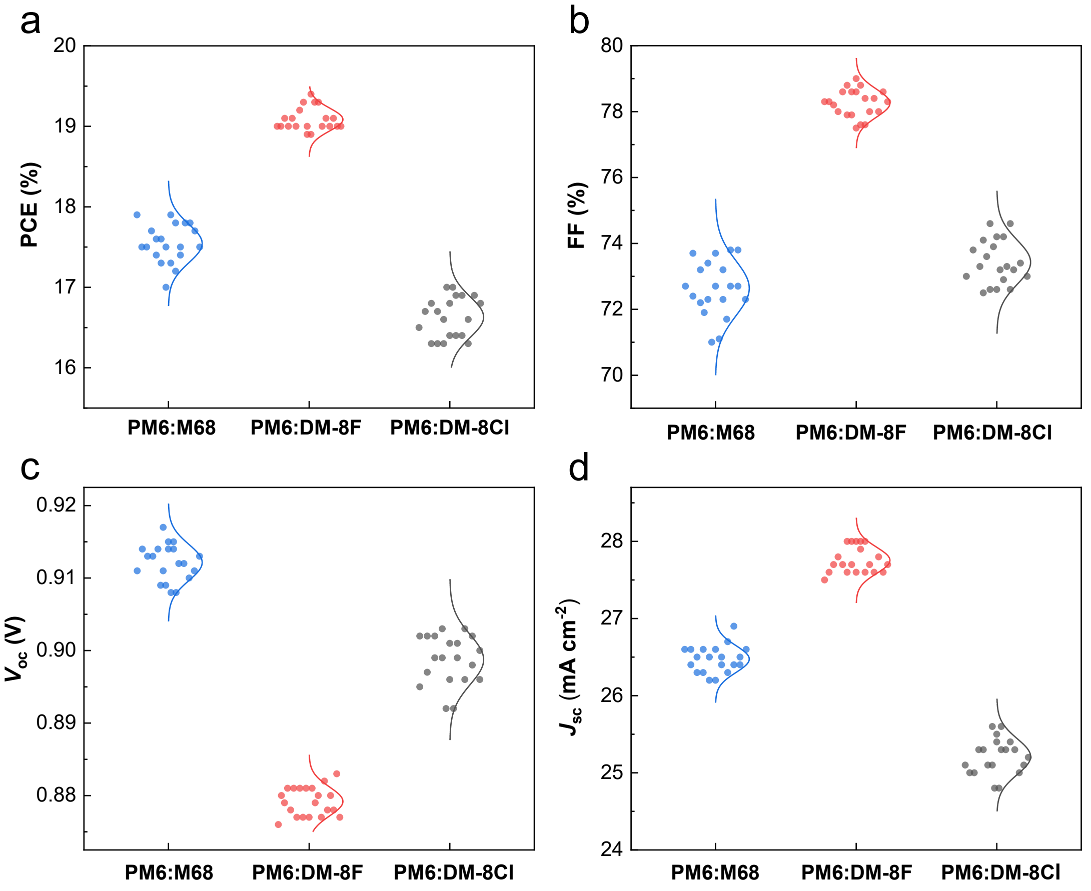

Supplementary Figure S4. Statistical distribution of key performance parameters for PSCs based on various acceptors, including (a) PCE, (b) FF, (c) V_{oc}, and (d) J_{sc}.

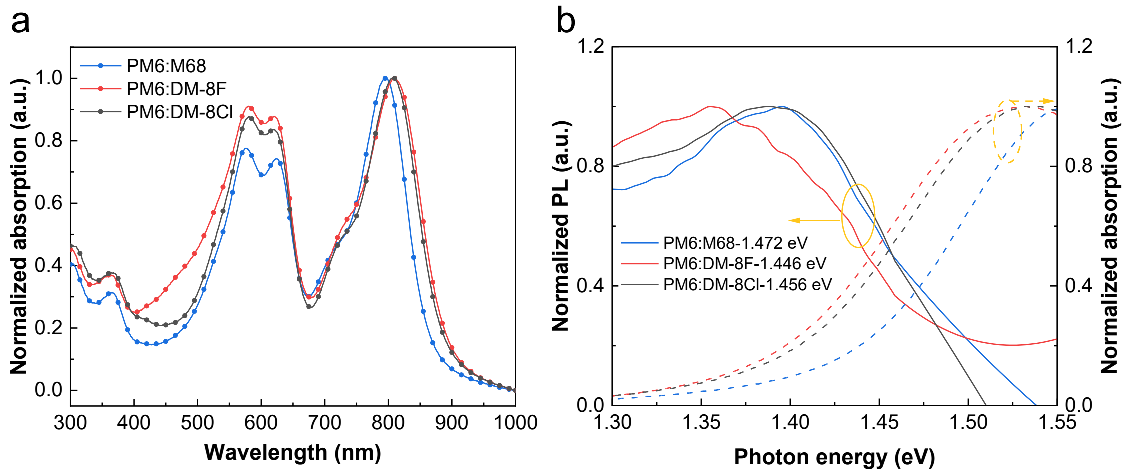

Supplementary Figure S5. (a) Normalized absorption spectra of three blend films on the wavelength scale. (b) The PL spectra (solid lines) and absorption spectra (dashed lines) of three blend films on the photon energy scale.

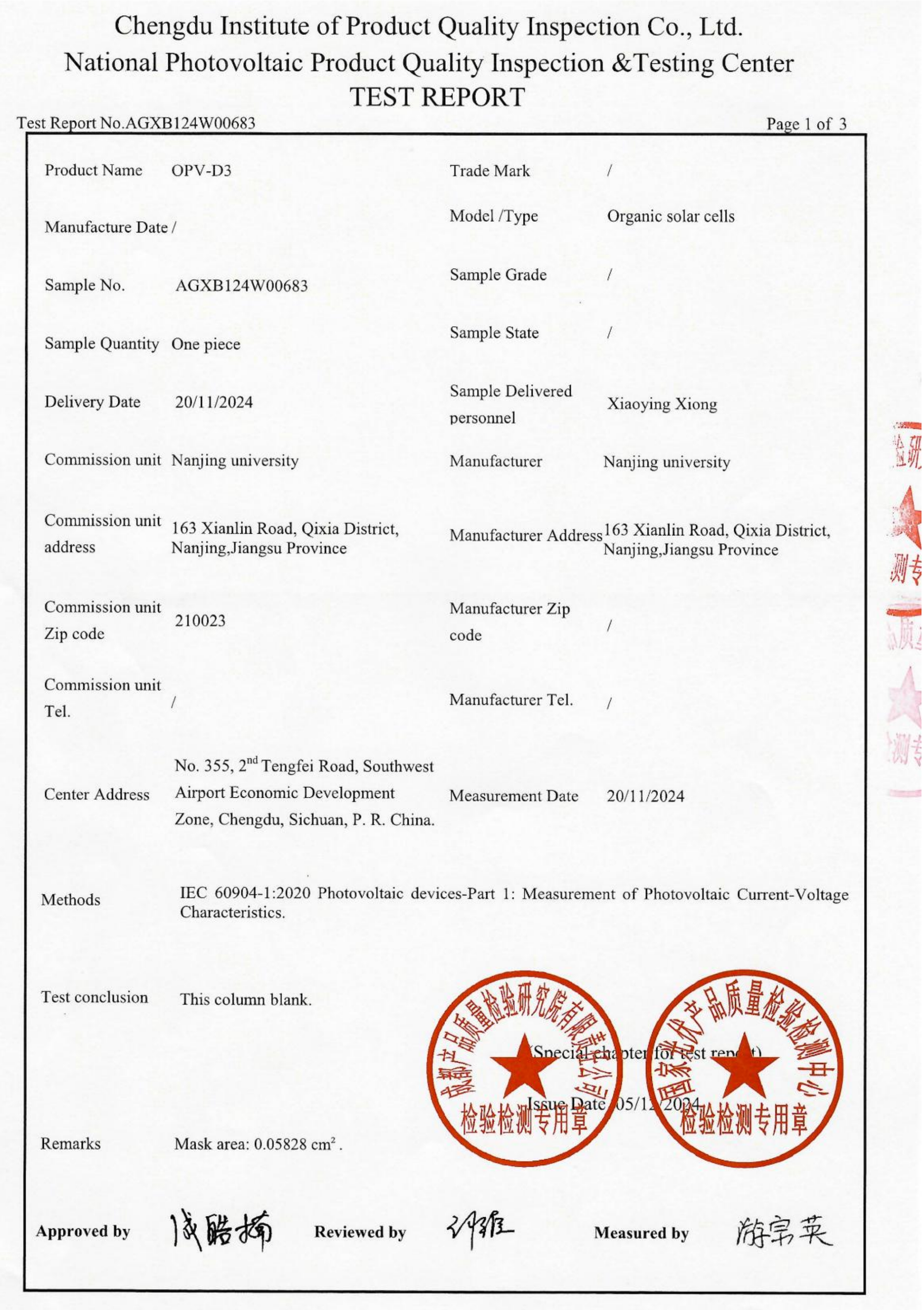

Chengdu Institute of Product Quality Inspection Co., Ltd.
National Photovoltaic Product Quality Inspection &Testing Center
TEST REPORT

Test Report No.AGXB124W00683 — Page 1 of 3

Product Name	OPV-D3	Trade Mark	/
Manufacture Date	/	Model /Type	Organic solar cells
Sample No.	AGXB124W00683	Sample Grade	/
Sample Quantity	One piece	Sample State	/
Delivery Date	20/11/2024	Sample Delivered personnel	Xiaoying Xiong
Commission unit	Nanjing university	Manufacturer	Nanjing university
Commission unit address	163 Xianlin Road, Qixia District, Nanjing,Jiangsu Province	Manufacturer Address	163 Xianlin Road, Qixia District, Nanjing,Jiangsu Province
Commission unit Zip code	210023	Manufacturer Zip code	/
Commission unit Tel.	/	Manufacturer Tel.	/
Center Address	No. 355, 2nd Tengfei Road, Southwest Airport Economic Development Zone, Chengdu, Sichuan, P. R. China.	Measurement Date	20/11/2024

Methods: IEC 60904-1:2020 Photovoltaic devices-Part 1: Measurement of Photovoltaic Current-Voltage Characteristics.

Test conclusion: This column blank.

(Special chapter for test report)

Issue Date 05/12/2024

Remarks: Mask area: 0.05828 cm^2.

Approved by **Reviewed by** **Measured by**

Supplementary Figure S6-1. Independent certification of a PM6:DM-8F-based OSC confirming a PCE of 19.20%.

Chengdu Institute of Product Quality Inspection Co., Ltd.
National Photovoltaic Product Quality Inspection &Testing Center
TEST REPORT

Test Report No.AGXB124W00683

Test Results（Forward scanning）:

No.	Test item(s)	Unit	Results
1	Current-voltage characteristics measurement	---	---
1.1	Open-circuit voltage, V_{OC}	V	0.874
1.2	Short-circuit current, I_{SC}	mA	1.612
1.3	Maximum-power, P_{max}	mW	1.119
1.4	Maximum-power voltage, $V_{p\text{-}max}$	V	0.760
1.5	Maximum-power current, $I_{p\text{-}max}$	mA	1.472
1.6	Fill factor, FF	%	79.40
1.7	Conversion efficiency, η	%	19.20

Current-voltage characteristics under STC

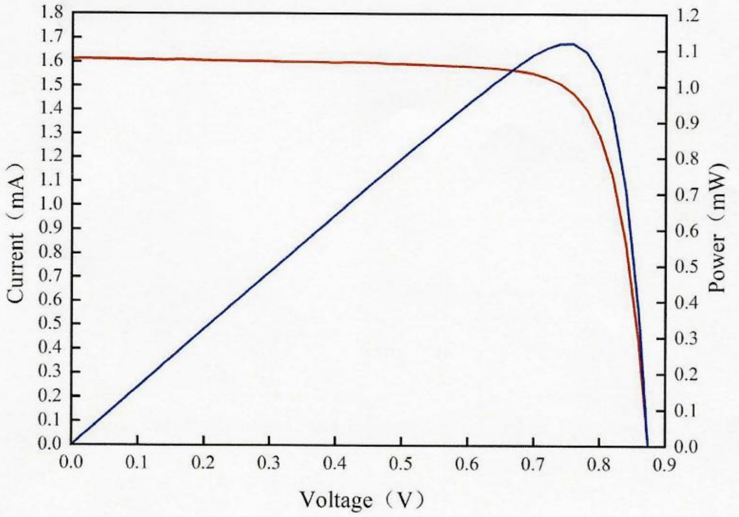

Remark: Sample was tested under the irradiation with a steady-state class calibrated AAA solar simulator (AM1.5-G 1000.0 W/m^2 basted on mono-Si reference cell) at 25±1 °C. Designated area defined by thin metal aperture mask.
The measuring uncertainty : U_{rel}(Pmax)=2.85%(k=2); U_{rel}(Isc)=2.69%(k=2); U_{rel}(Voc)=1.57%(k=2).

Supplementary Figure S6-2. Independent certification of a PM6:DM-8F-based OSC confirming a PCE of 19.20%.

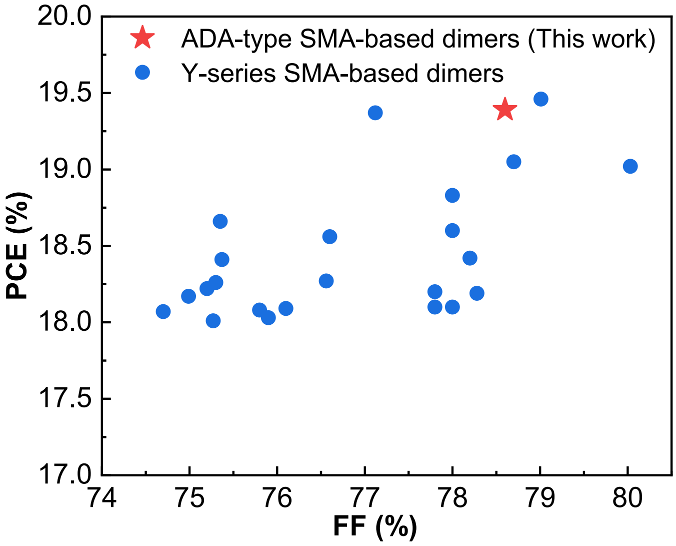

Supplementary Figure S7. Comparison of the photovoltaic performance of the ADA-type SMA-based dimerized acceptors developed in this work with previously reported Y-series SMA-based dimerized acceptors featuring an extended π-conjugated backbone (The original data are provided in Table S10).

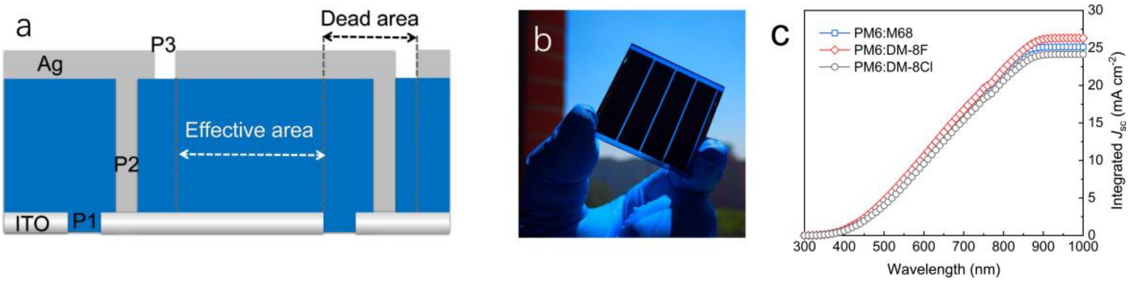

Supplementary Figure S8. (a) Schematic diagram of the module device structure featuring an effective active layer composed of PEDOT:PSS/PM6:DM-8F/PDIN. (b) The picture of a module device consisting of four subcells connected in series. (c) The integrated J_{sc} curves obtained from the EQE data of small-area devices employing PM6:M68, PM6:DM-8F and PM6:DM-8Cl active layers.

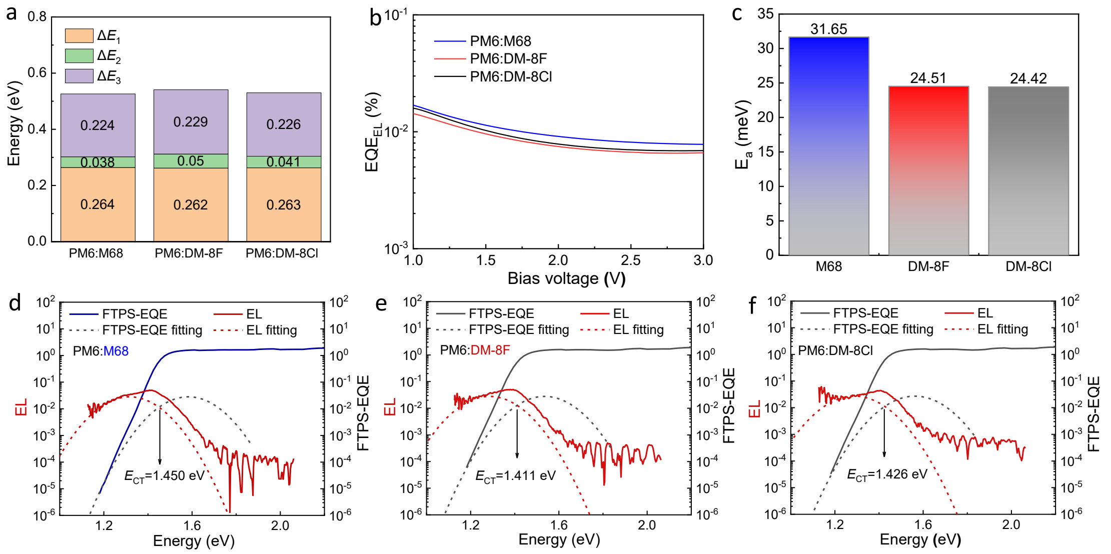

Supplementary Figure S9. (a) The detailed voltage losses of the PSCs based on PM6:M68, PM6:DM-8F and PM6:DM-8Cl. (b) EQE_{EL} for the PSCs. (c) Activation energy values of the acceptors. (d-f) EL sensitive and EQE spectra of the devices based on PM6:M68 (d), PM6:DM-8F (e) and PM6:DM-8Cl (f).

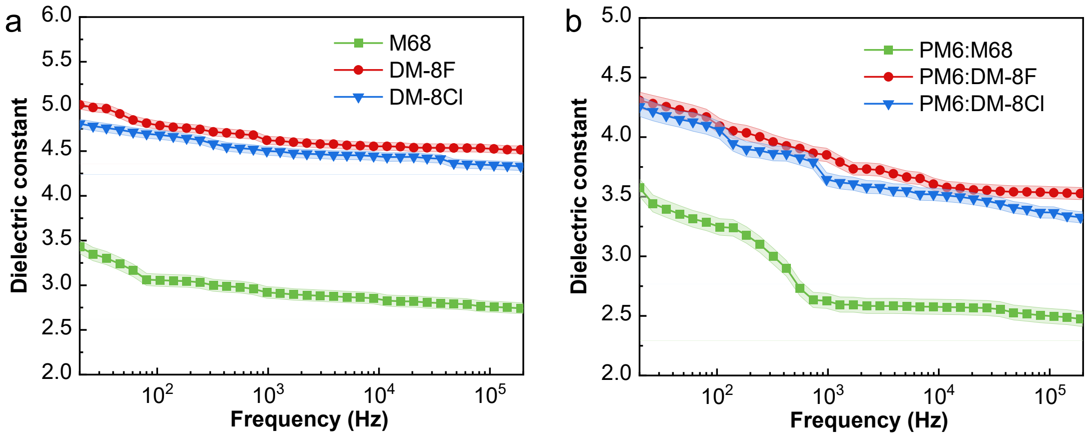

Supplementary Figure S10. (a) Capacitance *versus* frequency curves for M68, DM-8F, and DM-8Cl and (b) the corresponding blend films with PM6.

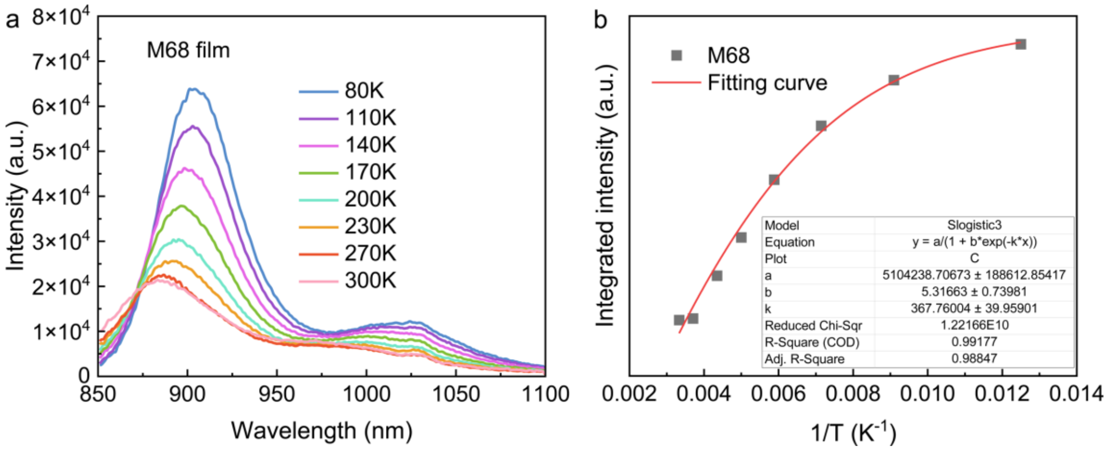

Supplementary Figure S11. (a) The temperature dependent PL spectra of the M68 film. (b) Integrated PL emission intensity of the M68 film as a function of temperature fitted by Arrhenius equation.

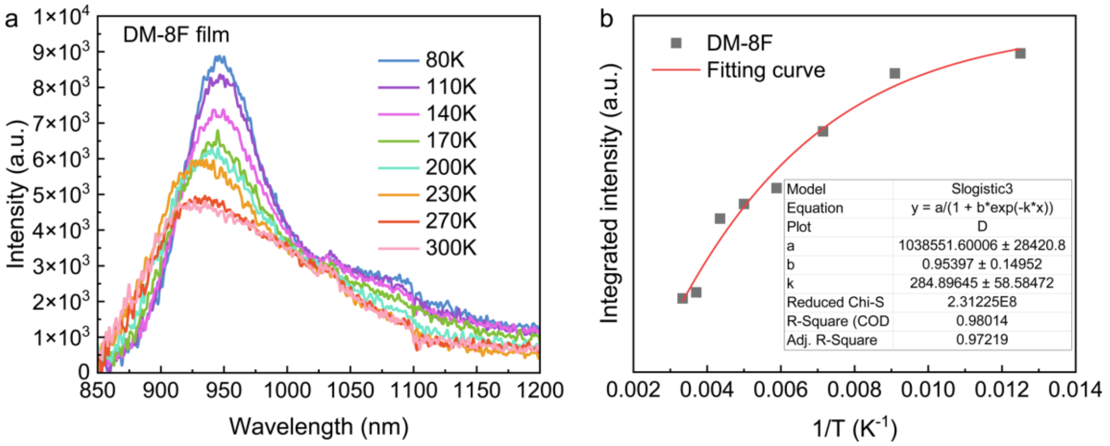

Supplementary Figure S12. (a) The temperature dependent PL spectra of the DM-8F film. (b) Integrated PL emission intensity of the DM-8F film as a function of temperature fitted by Arrhenius equation.

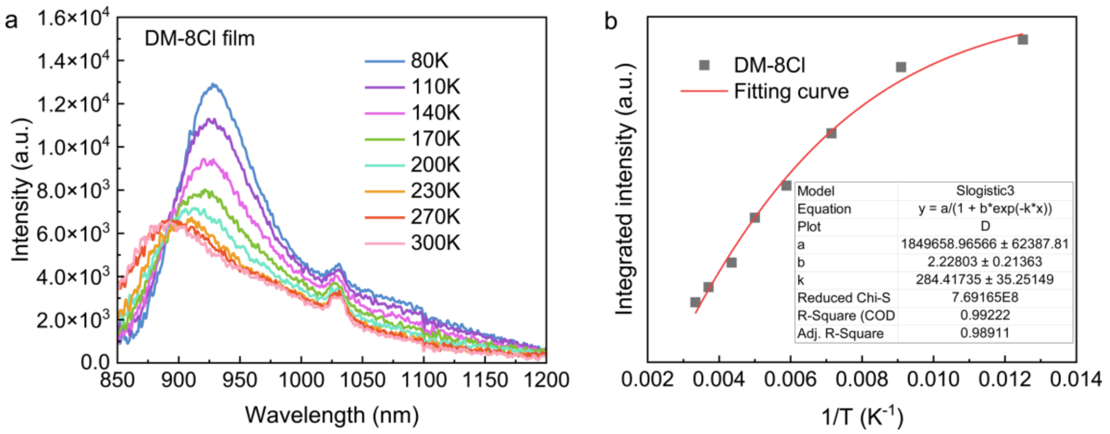

Supplementary Figure S13. (a) The temperature dependent PL spectra of the DM-8Cl film. (b) Integrated PL emission intensity of the DM-8Cl film as a function of temperature fitted by Arrhenius equation.

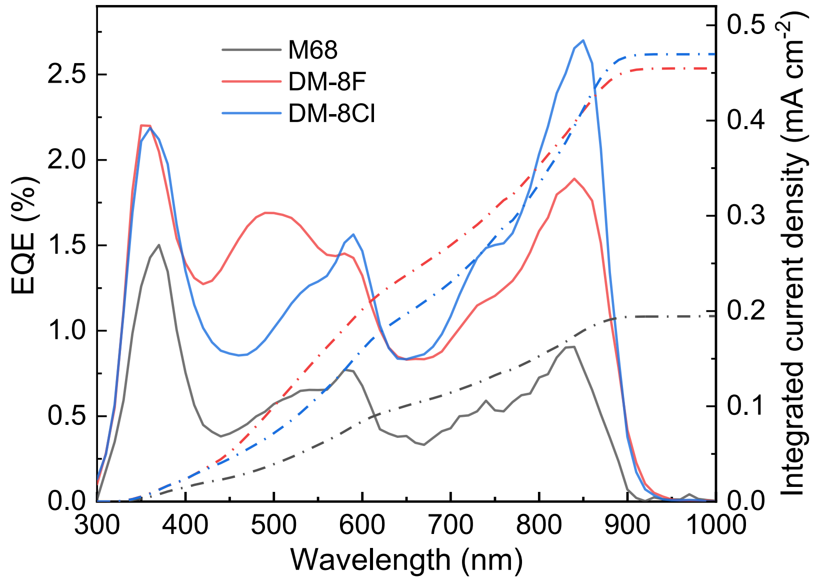

Supplementary Figure S14. EQE spectra (solid lines) and the corresponding integrated current density curves (dash lines) of photovoltaic devices based on pure acceptor materials.

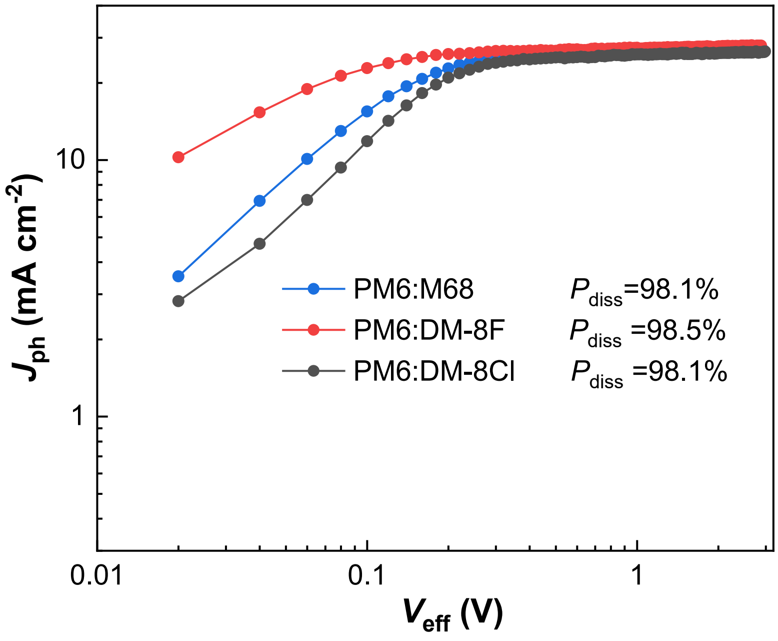

Supplementary Figure S15. Photocurrent density *versus* effective voltage (J_{ph}-V_{eff}) curves.

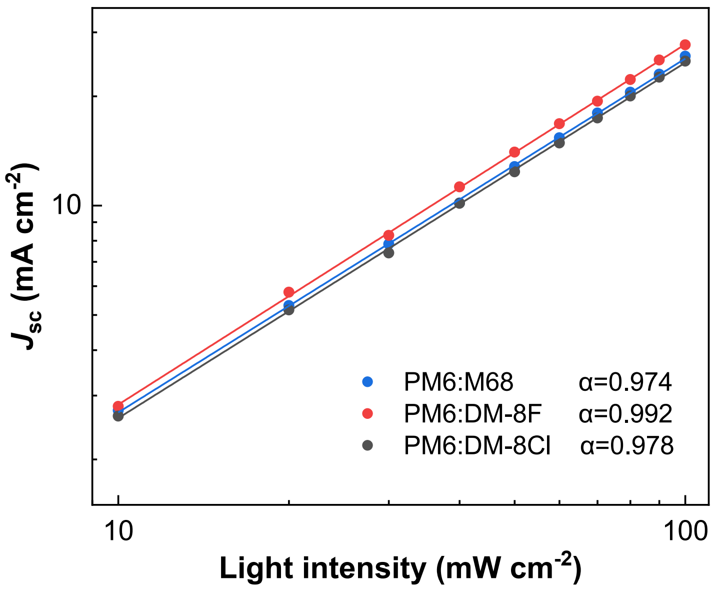

Supplementary Figure S16. Double logarithmic plots of J_{sc} as a function of incident light intensity.

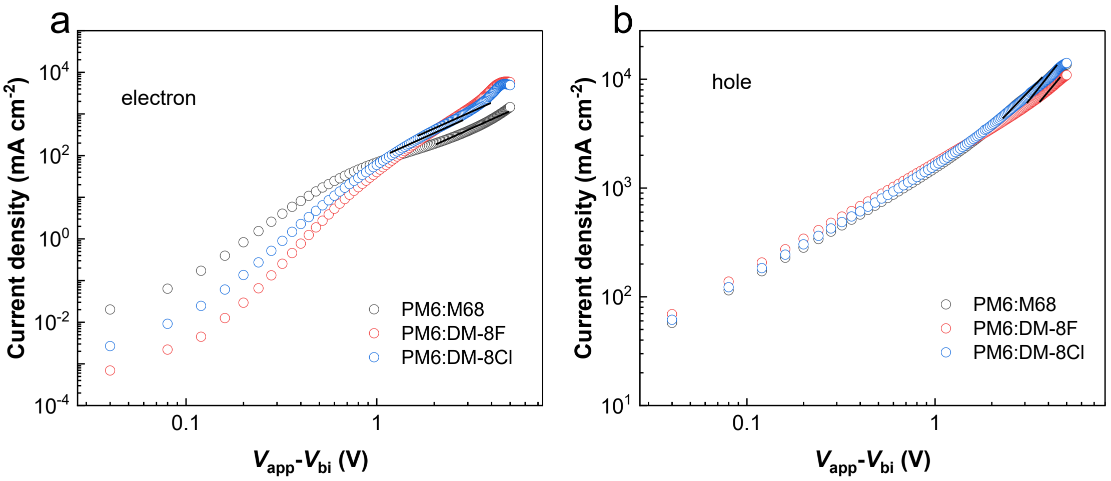

Supplementary Figure S17. *J-V* curves of (a) electron-only devices and (b) hole-only devices.

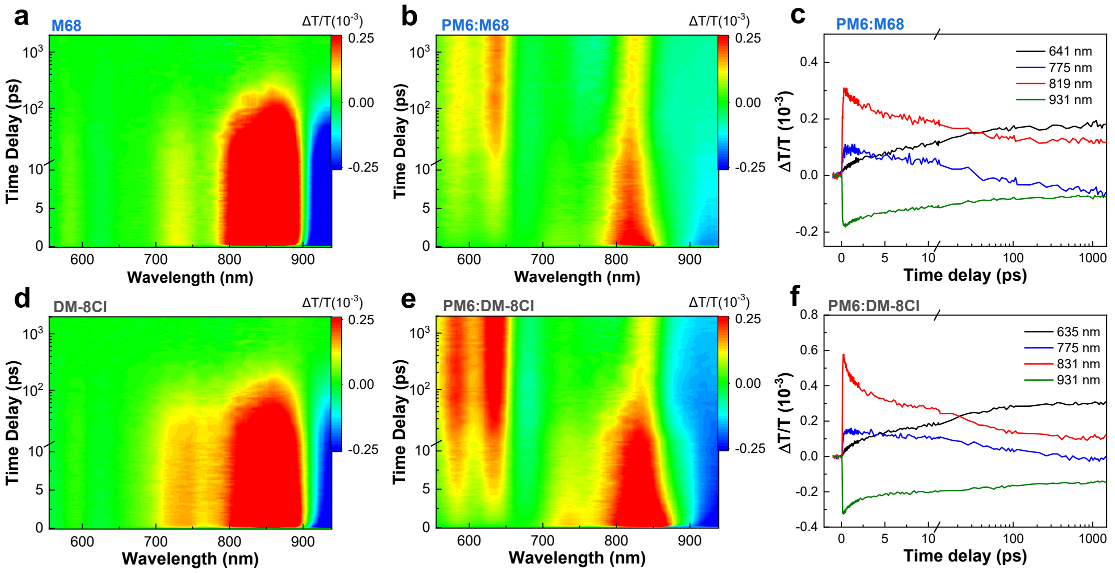

Supplementary Figure S18. The 2D TA spectra for (a) M68 neat film and (b) PM6:M68 blend film, (c) TA dynamics at different wavelengths for PM6:M68 blend film. The 2D TA spectra for (d) DM-8Cl neat film and (e) PM6:DM-8Cl blend film, (f) TA dynamics at different wavelengths for PM6:DM-8Cl blend film.

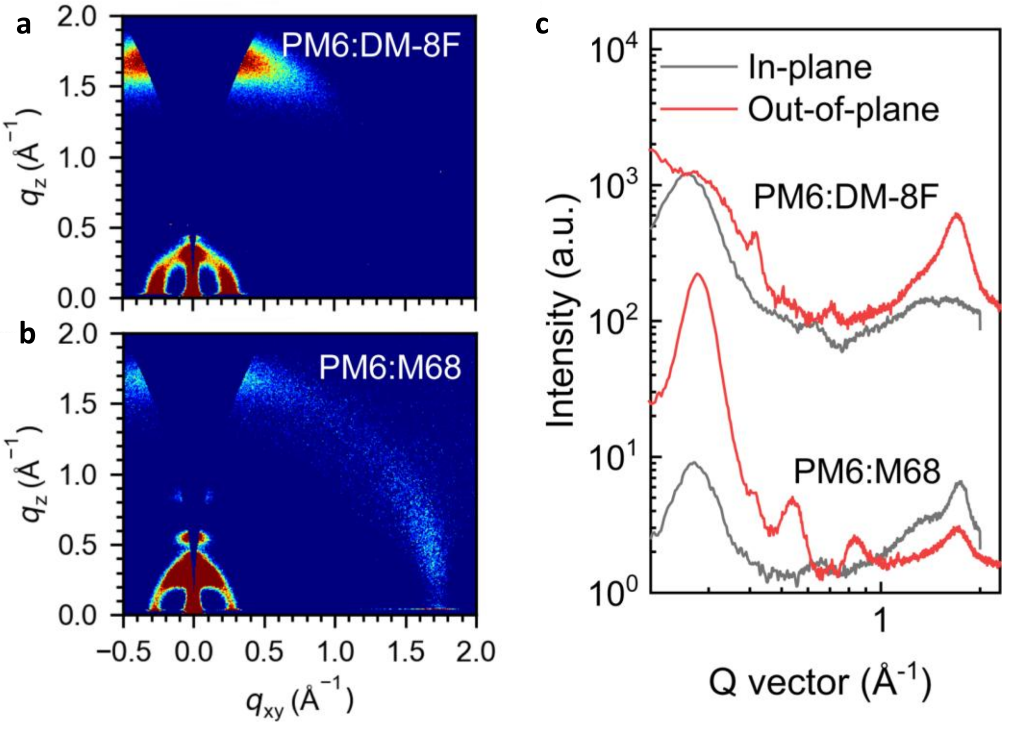

Supplementary Figure S19. (a, b) 2D GIWAXS patterns and (c) corresponding 1D line-cut profile of the blend film after thermal aging at 100 °C for 72 hours.

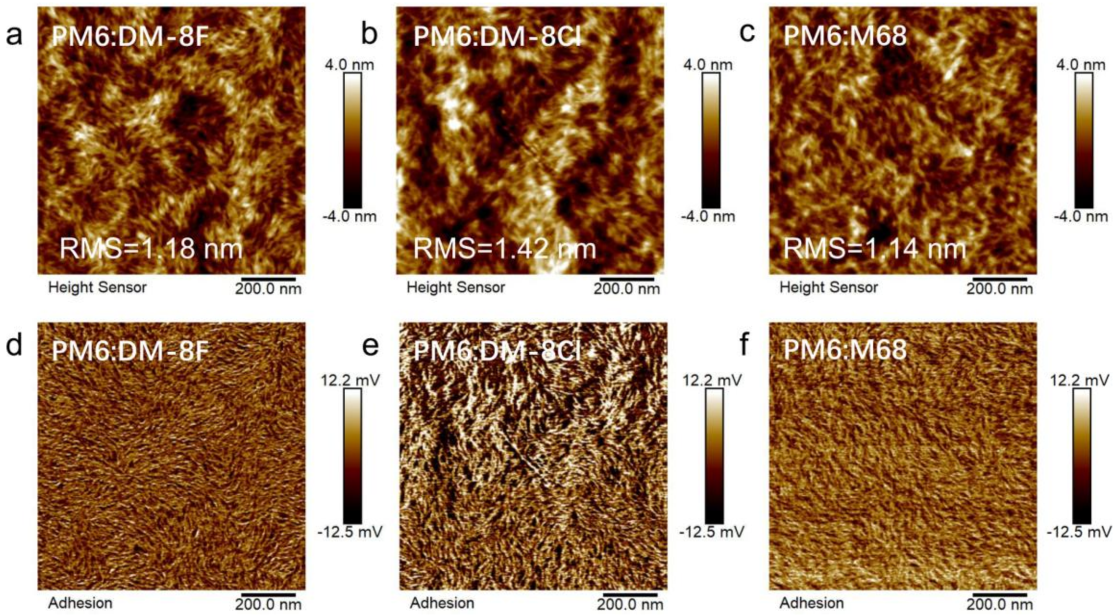

Supplementary Figure S20. AFM height (a-c) and phase (d-f) images of the active layers based on PM6:DM-8F, PM6:DM-8Cl, and PM6:M68.

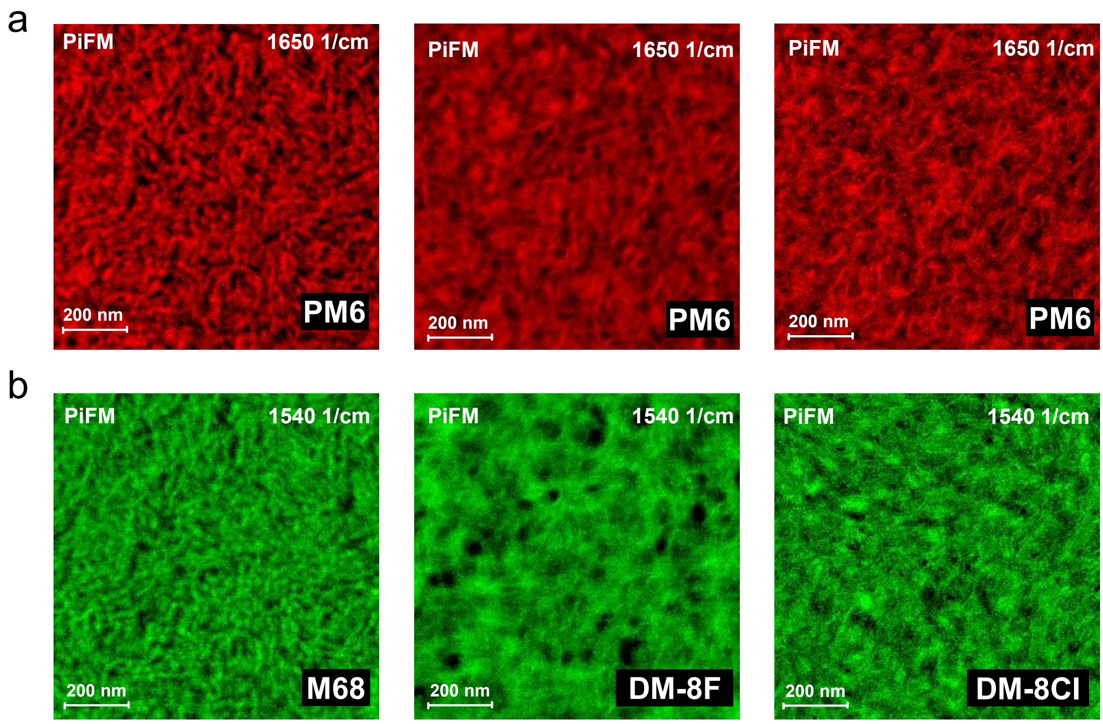

Supplementary Figure S21. PiFM images of PM6:acceptor blend films recorded at characteristic infrared (IR) wavenumbers: (a) 1650 cm^{-1}, corresponding to the donor PM6, and (b) 1540 cm^{-1}, associated with the acceptors M68, DM-8F, and DM-8Cl.

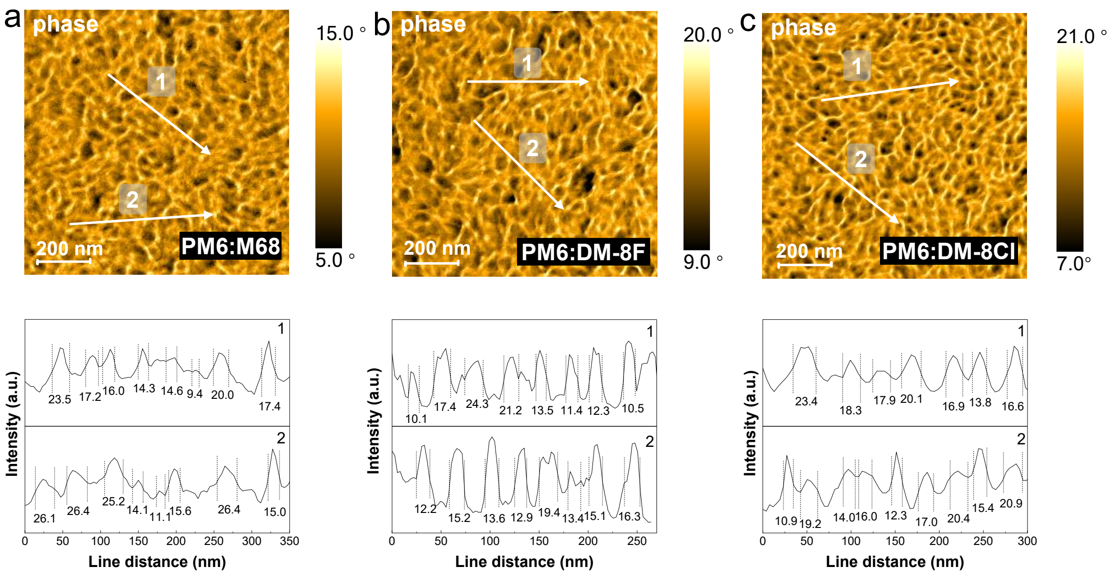

Supplementary Figure S22. Phase images of (a) PM6:M68, (b) PM6:DM-8F, and (c) PM6:DM-8Cl blend films, and the line profiles analyzed to determine the fibril widths (second row) for three blend films.

Supplementary Tables

Supplementary Table S1. Thermal stability, optical and electrochemical properties of M68, DM-8F and DM-8Cl.

NFA	T_d (°C)[a]	λ_{max}^{sol} (nm)	λ_{max}^{film} (nm)	FWHM (nm)[b]	ε_{max} (M cm^{-1})	HOMO/ LUMO (eV)[c]	PLQY (%)[d]
M68	307	743	822	93	2.31×10^5	-5.70/-3.95	11.35
DM-8F	312	767	828	137	4.48×10^5	-5.67/-3.97	1.54
DM-8Cl	314	769	817	118	4.34×10^5	-5.73/-4.02	4.42

[a] Decomposition temperature (T_d) obtained from thermo-gravimetric analysis. [b] full width at half maximum of the absorption peaks. [c] Measured by cyclic voltammetry. [e] Photoluminescence quantum yield (PLQY) tested by steady-state fluorescence spectrometer.

Supplementary Table S2. Summary of photovoltaic parameters for PM6:DM-8F-based conventional devices with different additives.

Additive[a-b]	V_{oc} (V)	J_{sc} (mA/cm^2)	FF (%)	PCE (%)
CN	0.871	27.0	78.1	18.4
2-CN	0.833	26.2	70.5	15.4
2-MN	0.903	21.7	67.8	13.3
2-EN	0.871	26.9	69.5	16.3
2-PN	0.832	26.3	68.4	15.0
TCB	0.871	27.1	76.7	18.1
DBCB	0.880	25.9	74.6	17.0
DCBB	0.879	26.1	74.6	17.1
TBB	0.876	26.1	73.8	16.9
BCB	0.875	26.0	74.4	16.9
DCB	0.875	25.0	76.9	16.8

[a]CN: 1-Chloronaphthalene; 2-CN: 2-Chloronaphthalene; 2-MN: 2-Methoxynaphthalene; 2-EN: 2-Ethoxynaphthalene; 2-PN: 2-Propoxynaphthalene; TCB: Trichlorobenzene; DBCB: 1,3-Dibromo-5-chlorobenzene; DCBB: 3,5-Dichlorobromobenzene; TBB: 1,3,5-Tribromobenzene; BCB: 4-Bromochlorobenzene; DCB: 1,4-Dichlorobenzene; [b]The additive was incorporated at a concentration of 0.5 vol%.

Supplementary Table S3. Summary of photovoltaic parameters for PM6:DM-8F-based conventional devices with different amounts of CN additive.[a-b]

CN (vol%)	V_{oc} (V)	J_{sc} (mA/cm^2)	FF (%)	PCE (%)
0	0.904	27.4	69.8	17.3
0.2	0.871	25.8	77.6	17.4
0.5	0.873	26.9	77.8	18.3
0.8	0.871	27.5	75	18.0

[a]The conventional device structure of ITO/2PACz/active layer/PNDIT-F3N/Ag; [b]The initial annealing temperature of the active layer was set at 100 °C and the duration of annealing was 10 minutes.

Supplementary Table S4. Summary of photovoltaic parameters for PM6:DM-8F-based conventional devices with different annealing times and temperatures.[a]

Temperature (°C)	Time (min)	V_{oc} (V)	J_{sc} (mA/cm^2)	FF (%)	PCE (%)
100	15	0.867	26.9	76.4	17.8
	20	0.874	27.2	76.3	18.1
	25	0.872	27.5	75.4	18.1
105	15	0.870	27.1	77.4	18.2
	20	0.872	27.2	77.4	18.4
	25	0.871	27.5	77.4	18.5
110	15	0.869	26.6	78.4	18.1
	20	0.870	27.1	76.8	18.1
	25	0.874	26.7	76.3	17.8

[a]The CN additive was added at a concentration of 0.5 vol%.

Supplementary Table S5. Summary of photovoltaic parameters for PM6:DM-8F-based conventional LBL devices with different film thicknesses.[a]

Thickness (nm)	V_{oc} (V)	J_{sc} (mA/cm^2)	FF (%)	PCE (%)
80	0.868	26.4	78.7	18.0
90	0.873	27.5	79.2	19.0
100	0.881	28.0	78.6	19.4
110	0.868	27.7	75.6	18.2

[a]The additive was incorporated at a concentration of 0.5 vol%, and the active layer was annealed at 105 ^{o}C for 25 minutes.

Supplementary Table S6. Summary of photovoltaic parameters for PM6:DM-8F-based BHJ conventional devices prepared with different solvents.[a]

Solvent	V_{oc} (V)	J_{sc} (mA/cm^2)	FF (%)	PCE (%)
Chloroform	0.871	27.6	78.3	18.8
Chlorobenzene	0.881	26.3	71.1	16.5

[a]The CN additive was incorporated at a concentration of 0.5 vol%, and the active layer was annealed at 105 ^{o}C for 25 minutes.

Supplementary Table S7. Summary of photovoltaic parameters for PM6:DM-8F-based BHJ conventional devices prepared with different concentrations in chloroform.[a]

Concentration	V_{oc} (V)	J_{sc} (mA/cm^2)	FF (%)	PCE (%)
14 mg/mL	0.875	26.9	78.2	18.4
15 mg/mL	0.871	27.6	78.3	18.8
16 mg/mL	0.881	26.7	76.0	17.9

[a]The CN additive was incorporated at a concentration of 0.5 vol%, and the active layer was annealed at 105 ^{o}C for 25 minutes.

Supplementary Table S8. Summary of photovoltaic parameters for PM6:DM-8F-based inverted devices prepared with different methods.[a-b]

Method	V_{OC} (V)	J_{SC} (mA/cm^2)	FF (%)	PCE (%)
BHJ	0.868	26.1	77.2	17.5
LBL	0.873	23.8	76.6	15.9

[a]The inverted device structure of ITO/ZnO/active layer/MoO_3/Ag; [b]The CN additive was incorporated at a concentration of 0.5 vol%, and the active layer was annealed at 105 °C for 25 minutes.

Supplementary Table S9. PCE and FF statistics of PSCs based on A-D-A-type SMAs and their dimerized acceptors with PCEs >12%.

Acceptor	Donor	PCE (%)	FF (%)	Ref.
DM-8F	PM6	19.39	78.6	**This work**
M36-FCl	PM6	18.51	78.0	*Energy Environ. Sci.* **2025**, *18*, 2895.
SN6C9-4F	PBDB-T	12.07	68.7	*Chin. J. Chem.* **2025**, *43*, 13.
TBB	PBQx-TF	16.2	74.0	*CCS Chem.* **2024**, *6*, 2749.
C-F	D18	15.4	77.2	*ACS Energy Lett.* **2024**, *9*, 1786.
S-F	D18	17.0	77.1	
PTBTT-4F	PM6	14.50	75.6	*Small* **2024**, *20*, 2305529.
PTBTT-4Cl	PM6	14.03	72.5	
TPBTT-4F	PM6	15.72	74.2	
TPBTT-4Cl	PM6	14.85	68.5	
DMT-HF	PM6	17.17	72.9	*Angew. Chem. Int. Ed.* **2024**, *50*, e202411155.
M36:PW-Se	PM6	18.00	77.3	*Adv. Mater.* **2024**, *36*, 2314169.
M36	PM6	18.2	78.1	*Adv. Energy Mater.* **2024**, *14*, 2401816.
MC7F3	PM1	17.61	79.5	*Chem* **2024**, *10*, 3131.
IMC8-4Cl	D18	13.99	62.5	*ACS Energy Lett.* **2024**, *6*, 2100.
BDOTP-1	D18-B	16.93	72.4	*Carbon Energy* **2023**, *5*, e250.
BDOTP-2	D18-B	15.48	71.1	
ZITI-N-6F	D18	16.11	73.8	*Fundam. Res.* **2025**, *5*, 2008.
ZITI-N-6F/ ZITI-N-8F	D18	17.09	75.9	
ZITI-N-8F	D18	15.20	73.6	
MD1T	PBDB-T	12.43	68.0	*Aggregate* **2023**, *4*, e322.
M36	PM6	17.02	78.4	*Nano Energy* **2023**, *107*, 108116.
MQ1-δ	PM6	12.08	63.3	*Chin. Chem. Lett.* **2023**, *34*, 108448.
ThPy6	PM6	16.11	78.9	*Adv. Funct. Mater.* **2022**, *32*, 2203200.
IDTP-4F	PM6	15.02	75.7	
TIT-2FIC	PM6	13.00	69.4	*Chem. Eng. J.* **2022**, *427*, 131674.
ThPy2	PM6	12.30	73.8	*Natl. Sci. Rev.* **2022**, *9*, nwac076.
ThPy3	PM6	15.30	77.1	

PTBTP-4F	PBDB-T	12.33	69.0	*Org. Electron.* **2022**, *103*, 106461.
2PIC	PM6	12.60	67.3	*Chin. J. Chem.* **2022**, *40*, 2861.
M14	PM6	16.46	76.6	*CCS Chem.* **2022**, *5*, 455.
M17	PM6	13.01	69.4	
MQ7-i	PM6	16.23	74.4	*J. Mater. Chem. A* **2022**, *10*, 23915.
cis-MF	J71	12.31	67.0	*Chem. Eng. J.* **2022**, *432*, 134393.
ML-2FM	PM6	15.33	73.4	*Solar RRL* **2022**, *6*, 2200119.
MC1	PM6	12.02	64.8	*Chem. Eng. J.* **2022**, *427*, 131022.
MS1	PM6	15.01	74.1	
M3	PM6	16.66	76.2	*Joule* **2021**, *5*, 197.
M6	PM6	15.45	70.3	*Adv. Funct. Mater.* **2021**, *31*, 2010436.
MQ5:M36	PM6	17.24	76.0	*ACS Appl. Mater. Interfaces,* **2021***, 13, 57684.*
M13	PM6	13.14	67.0	*Chem. Eng. J.* **2021**, *418*, 129497.
MQ3	PM6	13.51	66.9	*Angew. Chem. Int. Ed.* **2021**, *60*, 19314.
MQ5	PM6	15.64	74.3	
MQ6	PM6	16.39	75.7	
IT4F	PFBCPZ	15.3	78.5	*Nano Energy* **2021**, *82*, 105679.
DTTC-4Cl	T1	14.43	76.3	*Solar RRL* **2020**, *4*, 2000357.
DTSiC-4Cl	T1	14.46	73.6	
DTTC-4F	PM6	13.89	67.6	*J. Mater. Chem. A* **2020**, *8*, 1131.
DTTC-4Cl	PM6	15.42	74.0	
DTC-4F	PM6	13.37	70.4	
4TCIC-4F	PM7	13.02	73.0	*Solar RRL* **2020**, *4*, 1900417.
IPTBO-4Cl	PM6	15.00	72.6	*J. Mater. Chem. A* **2020**, *8*, 5458.
IPT-4F	PM6	14.96	74.2	
IDTP-4F	PM7	15.20	74.6	*Adv. Funct. Mater.* **2020**, *30*, 2000383.
TPIC-4Cl	PM7	15.31	75.5	*J. Mater. Chem. A* **2020**, *8*, 5927.
P6IC	PTB7-Th	12.20	70.2	*ACS Appl. Mater. Interfaces* **2020**, *12*, 14029.
M4	PM6	14.75	71.5	*J. Mater. Chem. A* **2020**, *8*, 24543.
M34	PM6	15.24	70.7	*Angew. Chem. Int. Ed.* **2020**, *59*, 21627.
M36	PM6	16.00	72.1	*Natl. Sci. Rev.* **2020**, *7*, 1886.
ZITI-N	J71	13.68	72.0	*iScience* **2019**, *19*, 883.
ZITI-C	J71	13.18	72.7	
CZTT-4F	PM6	12.07	65.1	*J. Mater. Chem. A* **2019**, *7*, 21903.
IT4F	T1	15.1	78.0	*Adv. Mater.* **2019**, *31*, 1808356.
IPIC-4Cl	PBDB-T	13.40	74.0	*Adv. Funct. Mater.* **2019**, *29*, 1903269.
INPIC-4F	PBDB-T	13.13	71.5	*Adv. Mater.* **2018**, *30*, 1707150.
SN6IC-4F	PBDB-T	13.20	73.0	*Chem. Mater.* **2018**, *30*, 5429.

Supplementary Table S10. PCE and FF statistics of PSCs incorporating Y-series SMA-based dimerized acceptors with extended π-conjugated backbones.

Acceptor	Donor	PCE (%)	FF (%)	Ref.
2BTP-2F-T	PM6	18.19	78.28	*Adv. Sci.*, **2022**, *9,* 2202513.
DYF-TF	D18	18.26	75.30	*CCS Chem.*, **2023**, *5,* 2576.
DYV	PM6	18.6	78	*ACS Energy Lett.*, ***2023***, *8,* 1344.
DYBO	PM6	18.08	75.8	*Joule*, ***2023***, *7,* 416.
EV-i	PM6	18.27	76.56	*Angew. Chem., Int. Ed.*, ***2023***, *62,* e202303551.
TDYα	PM6	18.1	78.0	*Nat. Commun.*, ***2023***, *14,* 2926.
DIBP3F-Se	PM6	18.09	76.1	*Angew. Chem. Int. Ed.*, ***2023***, *62,* e202302888.
DYA-I	D18	18.83	78	*Adv. Energy Mater.*, ***2023***, *13,* 2301283.
D-TPh	PM6	19.05	78.7	*Angew. Chem. Int. Ed.*, ***2024***, *63,* e202411044.
D-TN	PM6	18.42	78.2	
DY-TVCl	PM6	18.01	75.27	*Macromol. Rapid Commun.*, ***2024***, 2400433.
DYSe-1	PM6	18.56	76.6	*Adv. Energy Mater.*, ***2024***, *14,* 2400938.
DYSe-2	PM6	18.22	75.2	
V-DYIC-4F	PM6	18.10	77.8	*Adv. Sci.*, ***2024***, *11,* 2406772.
i-YT	PM6	18.03	75.9	*Angew. Chem. Int. Ed.*, ***2024***, *63,* e202415994.
FDY-m-TAT	PM6	18.07	74.7	*Angew. Chem. Int. Ed.*, ***2024***, *63,* e202403139.
CH8-6	PM6	18.2	77.8	*Energy Environ. Sci.*, ***2024***, *17,* 5719.
Dimer-2CF	PM6	19.02	80.03	*Adv. Mater.*, ***2023***, *36,* 2310046.
GMA-SSS	PM6	18.66	75.35	*Energy Environ. Sci.*, ***2024***, *17,* 9144.
GMA-SSeS	PM6	19.37	77.12	
GMA-SeSSe	PM6	18.17	74.99	
WD-6	PM6	18.41	75.37	*Natl. Sci. Rev.*, ***2025***, *12,* nwae409.
QD-1	PM6	19.46	79.01	*Energy Environ. Sci.*, ***2025***, *18,* 5356.

Supplementary Table S11. Detailed E_{loss} parameters of PM6:DM-8F-, PM6:DM-8Cl- and PM6:M68-based conventional devices.

Device	E_{CT} (eV)	EQE_{EL}	E_{loss} (eV)	ΔE_1 (eV)	ΔE_2 (eV)	ΔE_3 (eV)
PM6:DM-8F	1.411	1.43×10^{-4}	0.541	0.262	0.050	0.229
PM6:DM-8Cl	1.426	1.59×10^{-4}	0.530	0.263	0.041	0.226
PM6:M68	1.450	1.69×10^{-4}	0.526	0.264	0.038	0.224

Supplementary Table S12. Carrier mobilities of the hole-only or electron-only devices based on PM6:M68, PM6:DM-8F, and PM6:DM-8Cl blend films measured by the SCLC method.

Sample	μ_e ($\times 10^{-4}$ cm^2 V^{-1} s^{-1})	μ_h ($\times 10^{-4}$ cm^2 V^{-1} s^{-1})	μ_h/μ_e
PM6:M68	1.40 (1.22±0.13)	2.94 (2.70±0.19)	2.10
PM6:DM-8F	3.73 (3.57±0.14)	3.63 (3.50±0.10)	0.97
PM6:DM-8Cl	3.33 (3.17±0.12)	2.71 (2.48±0.14)	0.81

Supplementary Table S13. Detailed parameters of single exciton decay dynamic for the neat acceptor films.[a]

Sample	κ (ps^{-1})	α (nm^3 ps^{-1})	D (ps^{-1})	L_D (nm)
M68	0.0139	234.8	9.3	25.9
DM-8F	0.0041	111.3	4.4	33.1
DM-8Cl	0.0123	205.1	8.2	25.8

[a]*κ:* Intrinsic exciton decay rate constant; *α:* Bimolecular exciton annihilation rate constant; *D*: Exciton diffusion coefficient; L_D: Exciton diffusion lengths.

Supplementary Table S14. The parameters of diffraction peaks from GIWAXS line-cuts of out-of-plane and in-plane profiles.

Sample	π-π stacking		lamellar stacking	
	$d_{\pi\text{-}\pi}$ [Å][a]	CCL [Å] (FWHM)[b]	d_l [Å][a]	CCL [Å] (FWHM)[b]
M68	3.69	22.91 (0.247)	20.43	106.76 (0.053)
DM-8F	3.62	15.02 (0.377)	18.90	76.54 (0.074)
DM-8Cl	3.77	13.21 (0.428)	23.71	97.48 (0.058)
PM6:M68	3.69	21.63 (0.261)	21.48	92.25 (0.061)
PM6:DM-8F	3.68	19.21 (0.294)	21.67	71.83 (0.079)
PM6:DM-8Cl	3.73	17.56 (0.322)	21.48	75.12 (0.075)

[a]The (010) diffraction peak along the q_z axis, and the (100) diffraction peak along the q_{xy} axis; [b]The crystal coherence length (CCL) estimated from the Scherrer equation (CCL=$2\pi K$/FWHM, K=0.9), where FWHM is the full width at half maximum of the diffraction peak.

Supplementary References

[S1] Wang P, Zhu YH and Tao HX *et al.* Polymerizing ladder-type heteroheptacene-cored small-molecule acceptors for efficient all-polymer solar cells. *Chin J Polym Sci* 2023; **41**: 1018-26.

[S2] Ma YL, Cai DD and Wan S *et al.* Control over π-π stacking of heteroheptacene-based nonfullerene acceptors for 16% efficiency polymer solar cells. *Natl Sci Rev* 2020; **7**: 1886-95.

[S3] Ma YL, Cai DD and Wan S *et al.* Ladder-type heteroheptacenes with different heterocycles for nonfullerene acceptors. *Angew Chem Int Ed* 2020; **59**: 21627-33.

[S4] Ma YL, Zhang M and Wan S *et al.* Efficient organic solar cells from molecular orientation control of M-Series acceptors. *Joule* 2021; **5**: 197-209.

[S5] Li Y, Wang LX, Fu HT, Zheng QD. Piperazine-functionalized arylene diimides as electron transport layers for high-efficiency and stable organic solar cells. *Adv Funct Mater* 2025*;* **35**, 2419342.

[S6] Sun YM, Seo JH and Takacs CJ *et al.* Inverted polymer solar cells integrated with a low-temperature-annealed sol-gel-derived ZnO film as an electron transport layer. *Adv Mater* 2021; **23**, 1679-83.

[S7] Root SE, Alkhadra MA and Rodriquez D *et al.* Measuring the glass transition temperature of conjugated polymer films with ultraviolet–visible spectroscopy. *Chem Mater* 2017*;* **29**, 2646-54.

[S8] Ghasemi M Balar N and Peng Z *et al.* A molecular interaction–diffusion framework for predicting organic solar cell stability. *Nat. Mater.* 2021; **20**, 525-32.

[S9] Vandewal K, Benduhn J and Nikolis VC. How to determine optical gaps and voltage losses in organic photovoltaic materials. *Sustainable Energy & Fuels* 2018*;* **2**, 538-44 .

[S10] Hughes MP, Rosenthal KD andn Ran NA *et al.* Determining the Dielectric Constants of Organic Photovoltaic Materials Using Impedance Spectroscopy. *Adv Funct Mater* 2018*;* **28**, 1801542.

[S11] Koster LJA, Shaheen SE, and Hummelen JC. Pathways to a New Efficiency Regime for Organic Solar Cells. *Adv Energy Mater* 2012*;* **2**, 1246-53.

[S12] Chen Z, Yu C and Shum K *et al.* Photoluminescence study of polycrystalline $CsSnI_3$ thin films: Determination of exciton binding energy. *J Lumin* 2012; **132**, 345-9.

[S13] Wang W, Wang H and Tang X *et al.* Phenothiazine-based covalent organic frameworks with low exciton binding energies for photocatalysis. *Chem Sci* 2022*;* **13**, 8679-85.

[S14] Li G, Fu P and Yue Q *et al.* Boosting exciton dissociation by regulating dielectric constant in covalent organic framework for photocatalysis. *Chem Catal* 2022*;* **2**, 1734-47.

[S15] Cowan SR, Roy A and Heeger AJ. Recombination in polymer-fullerene bulk heterojunction solar cells. *Phys. Rev. B* 2010; **82**, 245207.

[S16] Firdaus Y, Le Corre VM and Karuthedath S *et al.* Long-range exciton diffusion in molecular non-fullerene acceptors. *Nat Commun* 2020; **11**, 5220.
